# Nutritional state-dependent changes in subcortical limbic structures in anorexia nervosa are linked to leptin, ghrelin, and neurofilament light

**DOI:** 10.1038/s41386-026-02435-w

**Published:** 2026-05-21

**Authors:** Marie-Louis Wronski, Klaas Bahnsen, Fabio Bernardoni, Maria Seidel, Arne Doose, Inger Hellerhoff, Milena Schimack, Jonas L. Steinhäuser, Giulia Lombardo, Johanna L. Keeler, David M. Poitz, Tjalf Ziemssen, Kerstin Weidner, Veit Roessner, Joseph A. King, Carmine M. Pariante, Stefan Ehrlich

**Affiliations:** 1 Translational Developmental Neuroscience Section, Division of Psychological and Social Medicine and Developmental Neurosciences, Faculty of Medicine, TU Dresden, Dresden, Germany; 2 Department of Psychological Medicine, Institute of Psychiatry, Psychology & Neuroscience, King’s College London, London, UK; 3 Neuroendocrine Unit, Department of Medicine, Massachusetts General Hospital and Harvard Medical School, Boston, MA USA; 4 German Center for Child and Adolescent Health (DZKJ), Partner Site Dresden/Leipzig, Dresden, Germany; 5 Centre for Research in Eating and Weight Disorders (CREW), Department of Psychological Medicine, Institute of Psychiatry, Psychology & Neuroscience, King’s College London, London, UK; 6 Institute for Clinical Chemistry and Laboratory Medicine, Faculty of Medicine, University Hospital Carl Gustav Carus, TU Dresden, Dresden, Germany; 7 Center of Clinical Neuroscience, Faculty of Medicine, University Hospital Carl Gustav Carus, TU Dresden, Dresden, Germany; 8 Department of Psychotherapy and Psychosomatic Medicine, Faculty of Medicine, University Hospital Carl Gustav Carus, TU Dresden, Dresden, Germany; 9 Department of Child and Adolescent Psychiatry, Faculty of Medicine, University Hospital Carl Gustav Carus, TU Dresden, Dresden, Germany

**Keywords:** Translational research, Neurotrophic factors, Nervous system

## Abstract

Anorexia nervosa (AN) involves limbic dysfunction, yet its neurobiology remains unclear. Examining morphological alterations in subcortical limbic regions across AN’s nutritional states and associations with neuroendocrine markers may improve understanding. We hypothesized that subcortical limbic volumes would be reduced in AN, normalize partially following short-term weight-restoration, and fully after long-term recovery. We examined whether leptin, ghrelin, neurofilament light (NFL), and brain-derived neurotrophic factor (BDNF) predict subcortical limbic dynamics. We studied 547 female individuals (aged 12–30 years): 168 with acute AN, 113 reassessed after ≈3 months of weight-restoration (>14% BMI increase), 80 long-term weight-recovered individuals, and 299 healthy controls. Ten subcortical limbic structures were segmented with FreeSurfer. Blood concentrations of leptin, ghrelin, NFL, and BDNF were measured. Group comparisons and marker associations were analyzed with mixed-effects models. Mediation was tested via regression-based mediation models. Most subcortical limbic volumes were reduced in AN (mean = −5.3%; Cohen’s *d*-range = 0.24–0.76) but normalized after short-term weight-restoration (longitudinal change: mean = +5.8%; *d*-range = 0.30–0.56), except residual hypothalamic reductions. Rising leptin mediated weight gain-related volumetric increases in the left inferior tubular hypothalamus (proportion mediated = 52.8%) and left (96.7%) and right (156.8%) nucleus accumbens. Declining ghrelin mediated volumetric increase of the right nucleus accumbens (77.8%). Decreasing NFL following weight-restoration predicted increases in two hypothalamic subunits, the fornix, and right nucleus accumbens (*d*-range = 0.56–0.98). BDNF was unrelated to limbic dynamics. Subcortical limbic alterations in AN exceed other mental disorders, highlighting neuroplasticity. Neuroendocrine recovery (leptin and ghrelin) may reverse, and reduced axonal damage (NFL) may indicate neurostructural alterations in homeostatic and reward-related regions, with prognostic and therapeutic relevance in AN.

## Introduction

Anorexia nervosa (AN) is an eating disorder (ED) primarily affecting girls and young women, characterized by body image disturbance, fear of weight gain, and weight-loss behaviors, typically via caloric restriction [1]. The acutely underweight state of AN is associated with pronounced reductions in cerebral gray and white matter that normalize with weight-restoration and remain stable after long-term recovery [2–4]. Similar trajectories have been demonstrated in the amygdala [5] and hippocampus [6] substructures, both components of the limbic system—a network involved in reward, homeostasis, and neuroendocrine regulation [7, 8] that is likely implicated in AN pathophysiology [9]. However, dynamic changes in subcortical limbic structures beyond the amygdala and hippocampus across AN stages and their endocrine and neuronal correlates remain poorly understood.

Limbic functioning is disrupted in AN, with diminished appetitive signaling and altered reward sensitivity, potentially reflecting altered neural circuits [9, 10]. Supporting evidence includes altered food-related striatal activation [11], reduced nucleus accumbens resting-state connectivity [12], and blunted hypothalamic glucose reactivity [13]. Hypothalamic volume also correlates positively with BMI [14].

Acute AN is characterized by several endocrine alterations [15], including suppressed leptin [16], an adipocyte-derived hormone signaling energy sufficiency and modulating feeding- and reward-related neural circuits [17, 18]. Hypoleptinemia has been linked to structural alterations in the amygdala, hippocampus, and thalamus [5, 6, 19] and to deteriorations in mood, appetite, and physical activity [16]. Leptin levels normalize with weight-restoration [16]. Ghrelin, primarily secreted by the gastrointestinal tract, stimulates food intake and contributes to reward-related aspects of eating [20, 21]. Elevated in acute AN, ghrelin decreases during weight-restoration [22] and modulates hypothalamic signaling [23], white matter connectivity, and reward-driven behavior [10, 24–26]. Together, leptin and ghrelin link metabolic signals to the mesolimbic dopaminergic system, projecting from the ventral tegmental area to the nucleus accumbens and other subcortical limbic structures [10, 18, 20, 21, 26, 27].

Beyond endocrine markers, neuronal markers may provide additional insight into neurobiological processes underlying altered limbic functioning in AN [9, 26]. Neurofilament light chain (NFL) is a structural protein of the neuronal cytoskeleton released into extracellular fluids following axonal damage and serves as a marker of neuronal injury [28, 29]. Elevated circulating NFL levels have been reported in acute AN and tend to decline with weight-restoration [30, 31], potentially mirroring neurostructural alterations associated with severe undernutrition [29]. In acute AN, NFL levels inversely correlate with cortical thickness and white matter volume reductions [32, 33]. Brain-derived neurotrophic factor (BDNF), a neurotrophin involved in neuronal survival and synaptic plasticity, as well as mesolimbic reward pathways, has also been implicated in AN pathophysiology and is thought to be reduced in acute AN [26, 34–36]. Furthermore, BDNF predicts volumetric increases in hippocampal subfields following weight-restoration [37]. Clarifying how leptin, ghrelin, NFL, and BDNF relate to limbic alterations across nutritional states of AN may advance understanding of its neurobiology and inform novel pharmacological treatments [10, 27, 38, 39].

Recent advances in automated segmentation of in vivo brain MRI now enable larger-scale investigation of additional histologically and functionally distinct subcortical limbic [40] and hypothalamic structures [41], including hypothalamic subregions, the basal forebrain, the fornix, mammillary bodies, and the nucleus accumbens. These regions integrate food-related emotions, reward processing, and signals from appetite-regulating hormones and neuronal factors [7, 10, 26, 27, 38]. Leptin, ghrelin, and BDNF exert central effects through receptors expressed in limbic and hypothalamic regions [42–44]. For example, leptin and ghrelin receptors are highly expressed in the arcuate nucleus of the inferior tubular hypothalamus, a key region initiating food intake [41–43, 45]. Leptin, ghrelin, and BDNF also act within the nucleus accumbens via their respective receptors, modulating dopaminergic signaling underlying reward processing [46–48]. In contrast, NFL reflects neuroaxonal damage and typically shows elevated concentrations and neurostructural associations in neurological disorders, with emerging evidence suggesting similar patterns in psychiatric conditions, including acute AN [29–31, 33].

To our knowledge, this is the first study examining a broad range of subcortical limbic structures in AN using combined cross-sectional and longitudinal designs: (1) comparisons of acute AN and long-term weight-recovered individuals with healthy controls and (2) longitudinal dynamics before and after a 3-month weight-restoration intervention. Only two smaller cross-sectional studies have investigated hypothalamic structure in AN: one reported reduced inferior tubular hypothalamus volume in 78 acute AN patients compared with controls [49], whereas another reported hypothalamic enlargement in 13 acute AN patients [50]. Neither study found associations with leptin or ghrelin, nor did they address subcortical limbic regions beyond the hypothalamus or biomarkers beyond appetite-related hormones [49, 50]. While leptin and ghrelin are well-established correlates of structural and functional brain changes related to starvation and recovery [5, 6, 10, 19, 25–27], emerging evidence suggests that NFL and BDNF also change dynamically across AN and may indicate neurostructural alterations [32, 33, 37].

Consistent with previous literature [2–6, 19], we hypothesized that subcortical limbic structures would show substantial volumetric reductions in acute AN, partial normalization following short-term weight-restoration, and full recovery after long-term weight-restoration. Building on longitudinal evidence that increasing leptin mediates volumetric increases in amygdala nuclei [5] and that increasing BDNF correlates with hippocampal normalization [37] during weight-restoration, we further expected longitudinal associations between subcortical limbic structures and circulating markers. Specifically, we hypothesized that increases in leptin and BDNF and decreases in ghrelin and NFL following weight-restoration would predict volumetric increases in subcortical limbic structures in AN, particularly hypothalamic subregions and the nucleus accumbens, which play central roles in homeostatic and reward processing and show high leptin, ghrelin, and BDNF receptor density [10, 21, 42–44, 46–48]. If these neurobiological factors are linked to the recovery of subcortical limbic alterations beyond the effects of weight gain alone, they may hold clinical relevance as diagnostic or prognostic biomarkers and potential therapeutic targets [27, 38].

## Methods

### Study design and participants

This observational cohort study did not require prospective clinical trial registration. It was approved by the Institutional Review Board of TU Dresden and conducted according to the Declaration of Helsinki. All participants (and legal guardians for minors) provided written informed consent or assent.

The sample comprised 547 girls and young women: 168 acutely underweight patients with AN at baseline (AN-TP1; aged 12–29 years), 80 long-term weight-recovered individuals with a history of AN (RecAN; aged 14–30 years; mean recovery duration ≈5 years), and 299 age-matched healthy controls (HC; aged 12–30 years). Only females were included, given the higher prevalence of AN in females ( ≈ 10–14:1) [51]; generalizability to males is unknown.

AN diagnosis was established using the expert form of the Structured Interview for Anorexia and Bulimia Nervosa (SIAB-EX) following DSM-5 criteria [52], requiring BMI <10^th^ age percentile (<18 years) or <17.5 kg/m² (≥18 years). Patients underwent behaviorally oriented inpatient nutritional rehabilitation at university child/adolescent psychiatry or psychosomatic medicine departments. Controlled weight gain, electrolyte monitoring, and supplementation prevented refeeding syndrome.

Of the 168 AN participants, 113 were reassessed after an average of 89 days of inpatient weight-restoration (AN-TP2). Weight-restoration required ≥10% BMI increase. All AN-TP2 participants in this study exceeded the threshold ( > 14% BMI increase). Missing TP2 assessments resulted from withdrawal of consent, loss to follow-up, early discharge, or new MRI contraindications. Baseline characteristics of AN participants with and without TP2 follow-up were largely comparable (except a 4-point IQ difference), suggesting minimal attrition bias (Supplementary Material SM 1.1, Table S1.1).

RecAN participants maintained BMI ≥10^th^ age percentile (<18 years) or ≥18.5 kg/m² (≥18 years) for ≥6 months, had regular menses, and reported no recent restrictive eating, binge eating, or purging. Psychoactive medication use was minimal (three AN and four RecAN taking SSRIs; one AN and one RecAN taking mirtazapine).

HC were recruited from middle schools, high schools, and universities. They were normal-weight, eumenorrheic, and exhibited normal eating behavior based on SIAB-EX. Exclusion criteria included lifetime BMI <10^th^ age percentile (<18 years) or <17.5 kg/m² (≥18 years), any psychiatric disorder assessed using the Mini International Neuropsychiatric Interview (M.I.N.I.) [53], and psychoactive medication.

Participant flow is shown in a CONSORT flow diagram (SM 1.7). Additional details appear in Table 1 and SM 1.1–1.2 (e.g., further exclusion criteria, AN-subtypes, duration of illness [DOI], and co-existing mental conditions in AN and RecAN).

**Table 1:** Descriptive statistics of the study sample.

Characteristic	Acute AN group pre/post treatment				Recovered group		Control group		Test statistics			Post hoc contrasts
	AN-TP1		AN-TP2		RecAN		HC					
	*n*	Mean ± SD	*n*	Mean ± SD	*n*	Mean ± SD	*n*	Mean ± SD	*F*_(b/w | w/n)_	*p*_(b/w | w/n)_	*η*_p_2_(b/w | w/n)_	Significant at FDR-*q* < 0.05
A. Demographic and clinical variables												
Age (years)	168	16.37 ± 3.04	113	16.34 ± 2.32	80	23.15 ± 3.79	299	19.27 ± 4.42	81.79 | 520.73	<0.001 | <0.001	0.23 | 0.82	AN-TP1 < HC (*q* < 0.001); AN-TP2 > AN-TP1 (*q* < 0.001); AN-TP2 < HC (*q* < 0.001); RecAN > HC (*q* < 0.001)
IQ	155	111.74 ± 12.12		n/a	80	111.06 ± 9.76	297	112.23 ± 9.93	0.42	0.659	<0.01	n/a
SES	155	3.36 ± 0.92		n/a	55	2.97 ± 0.96	214	3.22 ± 1.05	3.17	0.043	0.01	All nonsignificant
Hand preference	165	1.02 ± 2.63		n/a	80	1.16 ± 2.80	294	1.07 ± 2.66	0.07	0.930	<0.01	n/a
BMI (kg/m^2^)	168	14.69 ± 1.41	113	18.93 ± 1.13	80	20.95 ± 1.89	299	21.10 ± 2.17	659.65 | 1399.91	<0.001 | <0.001	0.71 | 0.93	AN-TP1 < HC (*q* < 0.001); AN-TP2 > AN-TP1 (*q* < 0.001); AN-TP2 < HC (*q* < 0.001)
BMI-SDS	168	−3.24 ± 1.24	113	−0.70 ± 0.60	80	−0.48 ± 0.62	299	−0.13 ± 0.66	769.29 | 1098.25	<0.001 | <0.001	0.72 | 0.88	AN-TP1 < HC (*q* < 0.001); AN-TP2 > AN-TP1 (*q* < 0.001); AN-TP2 < HC (*q* < 0.001); RecAN < HC (*q* < 0.001)
Minimal lifetime BMI (kg/m^2^)	168	14.29 ± 1.43		n/a	80	14.45 ± 1.60	276	19.94 ± 1.84	721.92	<0.001	0.73	AN-TP1 < HC (*q* < 0.001); RecAN < HC (*q* < 0.001)
EDI-2 core score	164	25.97 ± 7.01	109	24.42 ± 7.41	73	18.56 ± 6.39	279	14.93 ± 4.53	180.56 | 9.14	<0.001 | 0.003	0.38 | 0.05	AN-TP1 > HC (*q* < 0.001); AN-TP2 < AN-TP1 (*q* = 0.003); AN-TP2 > HC (*q* < 0.001); RecAN > HC (*q* < 0.001)
BDI-II total score	166	23.39 ± 11.10	108	14.48 ± 10.84	72	7.99 ± 8.38	279	4.32 ± 4.97	282.55 | 141.44	<0.001 | <0.001	0.49 | 0.40	AN-TP1 > HC (*q* < 0.001); AN-TP2 < AN-TP1 (*q* < 0.001); AN-TP2 > HC (*q* < 0.001); RecAN > HC (*q* < 0.001)
B. Blood-based protein markers												
Leptin (ng/mL)	142	1.55 ± 2.29	104	12.00 ± 7.28	71	9.36 ± 6.11	274	12.69 ± 8.73	n/a	n/a	n/a	n/a
Leptin-log_e_	142	−0.83 ± 2.00	104	2.27 ± 0.75	71	2.07 ± 0.56	274	2.32 ± 0.70	300.18 | 466.79	<0.001 | <0.001	0.51 | 0.63	AN-TP1 < HC (*q* < 0.001); AN-TP2 > AN-TP1 (*q* < 0.001)
Ghrelin (pg/mL)	122	1462.93 ± 673.97	56	801.73 ± 289.82	39	1070.46 ± 471.81	226	931.92 ± 416.81	n/a	n/a	n/a	n/a
Ghrelin^0.75^ residualized	122	48.71 ± 76.78	56	−31.05 ± 40.33	39	−1.43 ± 57.27	226	−18.36 ± 54.02	59.98 | 167.93	<0.001 | <0.001	0.23 | 0.75	AN-TP1 > HC (*q* < 0.001); AN-TP2 < AN-TP1 (*q* < 0.001)
NFL (pg/mL)	132	14.40 ± 14.75	81	12.84 ± 43.05	68	7.02 ± 2.31	269	7.87 ± 22.85	n/a	n/a	n/a	n/a
NFL-log_e_ residualized	132	0.43 ± 0.61	81	−0.04 ± 0.68	68	−0.03 ± 0.30	269	−0.19 ± 0.46	79.23 | 106.42	<0.001 | <0.001	0.23 | 0.45	AN-TP1 > HC (*q* < 0.001); AN-TP2 < AN-TP1 (*q* < 0.001); AN-TP2 > HC (*q* = 0.003)
BDNF (ng/mL)	87	21.20 ± 5.84	61	21.43 ± 6.06	40	20.79 ± 5.25	171	21.28 ± 5.70	n/a	n/a	n/a	n/a
BDNF-log_e_ residualized	87	−0.01 ± 0.28	61	−0.01 ± 0.29	40	0.01 ± 0.26	171	<0.01 ± 0.26	0.24 | 0.10	0.785 | 0.757	<0.01 | <0.01	n/a
C. Head size												
eTIV (mm^3^)	168	1,493,633 ± 123,404		n/a	80	1,494,997 ± 108,237	299	1,507,141 ± 105,210	0.93	0.393	<0.01	n/a
D. Gray matter volumes												
Total gray matter volume (mm^3^)	168	645,308 ± 51,700	113	684,835 ± 54,253	80	659,609 ± 46,072	299	683,179 ± 53,440	139.68 | 373.87	<0.001 | <0.001	0.32 | 0.69	AN-TP1 < HC (*q* < 0.001); AN-TP2 > AN-TP1 (*q* < 0.001)
Total subcortical gray matter volume (mm^3^)	168	57,893 ± 4576	113	60,050 ± 4503	80	59,068 ± 4277	299	60,484 ± 4278	44.40 | 222.16	<0.001 | <0.001	0.14 | 0.63	AN-TP1 < HC (*q* < 0.001); AN-TP2 > AN-TP1 (*q* < 0.001)

All study participants were female and identified as European. In AN-TP1, 137 (81.55%) were <18 years vs. 31 (18.45%) ≥18 years; in RecAN, 8 (10.00%) were <18 years vs. 72 (90.00%) ≥18 years; in HC, 151 (50.50%) were <18 years vs. 148 (49.50%) ≥18 years. In AN-TP1, the mean ± SD age at first AN onset was 14.5 ± 2.8 years and the mean ± SD duration of the current AN episode (DOI) was 13.8 ± 17.8 months. AN-subtype was determined via SIAB-EX: 146 (86.90%) were restrictive and 22 (13.10%) binge/purge. In RecAN, the mean ± SD age at first AN onset was 14.7 ± 2.0 years and the mean ± SD duration of recovery from the former AN episode was 60.7 ± 42.1 months. Regarding (history of) co-existing mental conditions in AN-TP1, 13 had a depressive disorder, 11 an anxiety disorder, 1 post-traumatic stress disorder, 6 obsessive-compulsive disorder, 2 an adaptation disorder, 3 a personality disorder, 1 a developmental disorder, 1 Tourette syndrome, and 1 a somatization disorder. In RecAN, 23 had a depressive disorder, 6 an anxiety disorder, 1 post-traumatic stress disorder, and 2 obsessive-compulsive disorder. Selective serotonin reuptake inhibitors (SSRIs) were taken by 3 AN-TP1, 2 AN-TP2, and 4 RecAN; mirtazapine by 1 AN-TP1, 1 AN-TP2, and 1 RecAN. Current cigarette smoking ( > 1 cigarette/day on average) was reported by 14 RecAN and 8 HC. IQ was estimated with short versions of the German adaptation of the Wechsler Adult Intelligence Scale (WIE) [104]; or the Wechsler Intelligence Scale for Children (HAWIK-IV) [105] for participants aged ≤15 years. SES was estimated according to the participant’s (if >18 years) or the parental/household educational level and occupation group (range: 0 [lowest, leaving school without graduation] to 5 [highest, graduating from university]) [106]. Hand preference total score was assessed using items of the Annett Scale of Hand Preference (range: 0–12, smaller values indicate right-side preference/larger values left-side preference) [107]. Head size (C.) and gray matter volumes (D.) were extracted from FreeSurfer version 7.1.1 segmentation statistics [63]. IQ was estimated with short versions of the German adaptation of the Wechsler Adult Intelligence Scale (WIE) [104]; or the Wechsler Intelligence Scale for Children (HAWIK-IV) [105] for participants aged ≤15 years. SES was estimated according to the participant’s (if >18 years) or the parental/household educational level and occupation group (range: 0 [lowest, leaving school without graduation] to 5 [highest, graduating from university]) [106]. Hand preference total score was assessed using items of the Annett Scale of Hand Preference (range: 0–12, smaller values indicate right-side preference/larger values left-side preference) [107]. Head size (C.) and gray matter volumes (D.) were extracted from FreeSurfer version 7.1.1 segmentation statistics [63]. Number of participants (*n*), mean value, and standard deviation (SD) are stated for each variable and study group. A./C. Group differences were tested using general linear models (GLMs) for single-measure variables (n/a at AN-TP2) and linear mixed effects (LME) models for repeated-measures variables (see SM 1.6 for details). B. Marker concentrations were transformed to approximate normality: leptin was log_e_-transformed, ghrelin was power(¾)-transformed (*x*^0.75^ given mild positive skewness), NFL was log_e_-transformed, and BDNF was log_e_-transformed. In case of significant batch and/or storage time effects (SM 1.4), marker concentrations were subsequently residualized using multiple linear regression (SM 1.4, Table S1.4). Group differences in marker concentrations were tested using LME models, adjusted for age. D. Group differences in gray matter volumes were tested using LME models, adjusted for age, age [2], and eTIV. As test statistics for A.–D., *F*-value (between-participants [b/w], i.e., AN-TP1-RecAN-HC | within-participant [w/n], i.e., AN-TP2-AN-TP1), two-tailed *p* value, and effect size estimate partial *η*² are reported. Significant (*q* < 0.05) post hoc pairwise contrasts are stated following Benjamini-Hochberg false discovery rate (FDR)-adjustment [69] of *p* values across all contrasts of interest per variable.

*AN* participants with acute anorexia nervosa in the acutely underweight state (TP1) and reassessed after short-term weight-restoration (TP2), *BDI-II total* total score of Beck Depression Inventory-II, *BDNF* brain-derived neurotrophic factor, *BMI(-SDS)* body mass index(-standard deviation score), *EDI-2 core* averaged score comprising the core subscales drive for thinness, body dissatisfaction, and bulimia of Eating Disorder Inventory-2, *eTIV* estimated total intracranial volume, *HC* healthy control participants, *IQ* intelligence quotient, *lh* left hemisphere, *NFL* neurofilament light, *RecAN* long-term weight-recovered participants with a history of AN, *rh* right hemisphere, *SES* socioeconomic status.

### Clinical measures

BMI standard deviation scores (BMI-SDS), adjusted for age and sex [54, 55], were calculated. Core ED symptoms were assessed with the Eating Disorder Inventory-2 (EDI-2) [56] using the mean of the drive for thinness, body dissatisfaction, and bulimia subscales [5]. Depressive symptoms were measured with the Beck Depression Inventory-II (BDI-II) [57]. Data were managed using REDCap [58].

### Blood-based endocrine and neuronal markers

Plasma leptin, plasma total ghrelin (hereafter ghrelin), serum NFL, and serum free BDNF were measured in fasting venous blood collected between 7–9 a.m. (AN-TP1 within 96 h after treatment initiation). Samples were immediately processed (SM 1.3).

Leptin (single measurement), ghrelin (duplicate), and BDNF (single or duplicate depending on batch; Table S1.4) were measured using ELISA assays (leptin: BioVendor Research and Diagnostic Products, Brno/Czech Republic; ghrelin: Millipore-Sigma [Merck], Burlington/MA/USA; BDNF: R&D Systems, Minneapolis/MN/USA). NFL was quantified in duplicate using the digital single-molecule array SIMOA™ Human Neurology 4-Plex assay and HD-1 Analyzer (Quanterix, Lexington/MA/USA), providing higher sensitivity than conventional assays [59]. When duplicates were available, means were used.

Manufacturer-reported leptin intra- and inter-assay coefficients of variation (CVs) were <6%. For ghrelin, NFL, and BDNF, sample-derived intra-assay CVs were ≈5%, ≈7%, and ≈6%, respectively (manufacturer-reported inter-assay CVs <8%, <11%, and <12%). Measurements with intra-assay CV > 20% were excluded (ghrelin: *n* = 10; NFL: *n* = 10; BDNF: *n* = 1). Marker distributions were normalized using natural log-transformation (leptin, NFL, BDNF) and a power-transformation (ghrelin, *x*^0.75^).

Leptin values below the assay detection limit in AN-TP1 (*n* = 39) were imputed using censored likelihood multiple imputation [60], which preserves their natural variability (SM 1.3). Below detection limit values did not occur for ghrelin, NFL, and BDNF. Marker concentrations were residualized when batch or storage time effects were detected (ghrelin and NFL adjusted for batch, BDNF for batch and storage time [37, 61, 62]; SM 1.4, Table S1.4).

### Subcortical limbic structures: MRI data processing

**Fig. 1:**
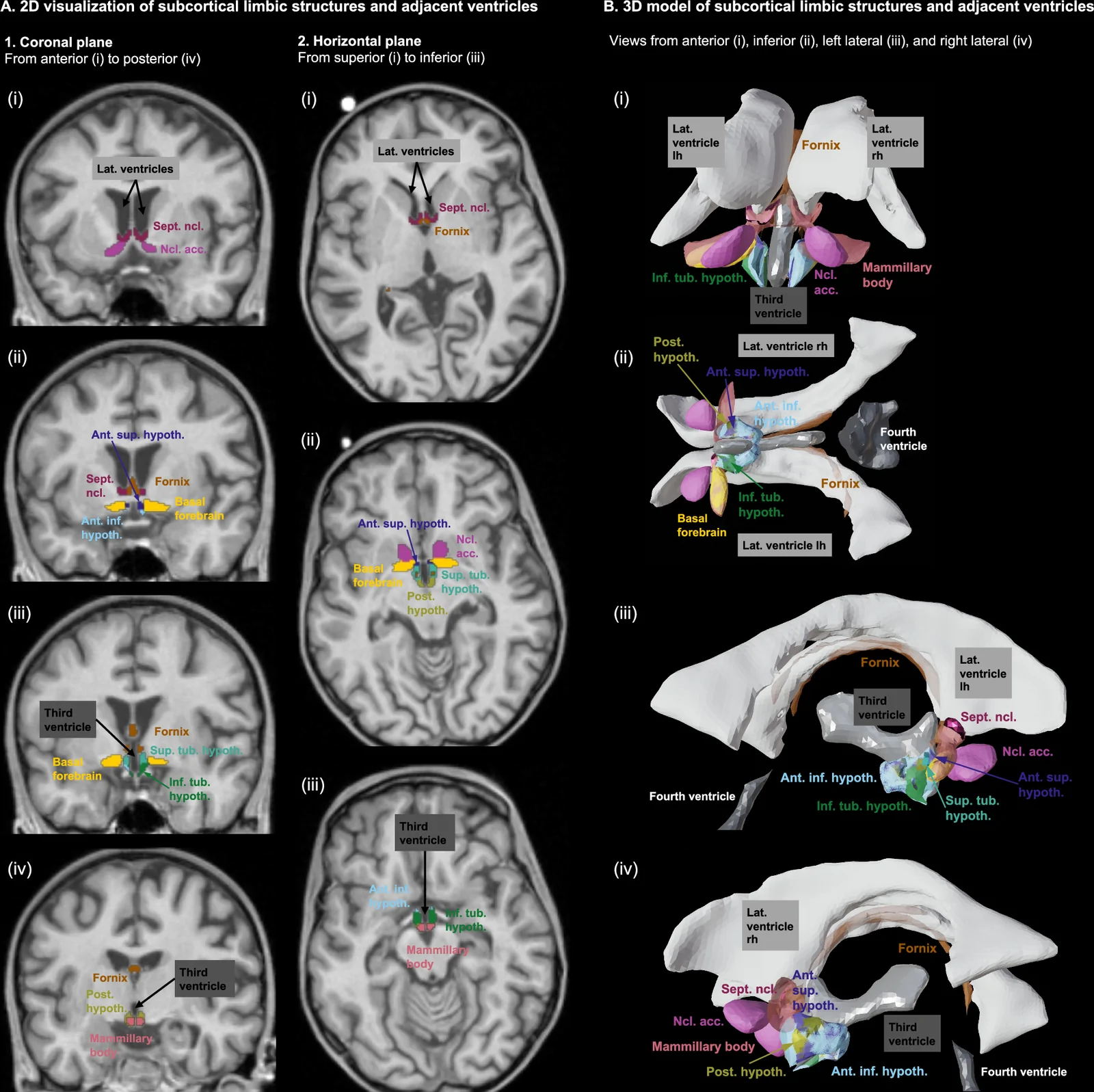
Two- and three-dimensional visualizations of subcortical limbic structures. Ant. inf. hypoth. anterior inferior hypothalamus, ant. sup. hypoth. anterior superior hypothalamus, inf. tub. hypoth. inferior tubular hypothalamus, lat. lateral (ventricle), lh left (brain) hemisphere, ncl. acc. nucleus accumbens, post. hypoth. posterior hypothalamus, rh right (brain) hemisphere, sept. ncl. septal nucleus, sup. tub. hypoth. superior tubular hypothalamus. MRI parameters (T1-weighted): MP-RAGE (magnetization prepared rapid acquisition gradient echo) sequence with 176 sagittal slices (1 mm thickness, no gap), TR = 1900 ms, TE = 2.26 ms, flip angle = 9˚, resolution = 1.0 × 1.0 × 1.0 mm^3^, FoV = 256 × 224 mm^2^, bandwidth = 200 Hz/pixel. **A** 2D FreeView [63] snapshots in different planes and **B** 3D model in different perspectives (created with Blender [108]) of the subcortical limbic structures analyzed in this study (and adjacent ventricles). **A**, **B** were obtained from a scan of a study participant after FreeSurfer version 7.1.1-based subcortical limbic segmentation [40] and hypothalamic subsegmentation [41]. To enhance accessibility, color blindness-friendly labeling was used in all figures (https://personal.sron.nl/~pault/data/colourschemes.pdf).

All participants were scanned between 8–9 a.m. (fasting; same day as blood sampling; AN-TP1 within 96 h after treatment initiation) at a single site using the same 3.0 T scanner (Magnetom Trio, Siemens, Erlangen/Germany). High-resolution 3D T1-weighted images were acquired using a magnetization-prepared rapid acquisition gradient-echo sequence (see Fig. 1 for details). MRI data were preprocessed with the FreeSurfer (v7.1.1) recon-all pipeline [63] for automated cortical reconstruction and subcortical segmentation.

Subcortical limbic structures (Fig. 1)—hypothalamic subregions (anterior superior/inferior, superior/inferior tubular, posterior [excluding mammillary bodies]), basal forebrain, fornix, mammillary body, nucleus accumbens, and septal nucleus—were segmented bilaterally using FreeSurfer processing streams *ScLimbic* [40] and *Segmentation of hypothalamic subunits* [41]. Both use deep learning models trained on manually labeled T1-weighted scans with augmentation to increase segmentation robustness [40, 41].

Quality control (QC) followed ENIGMA standards [64] using a multi-stage procedure. After assessing general image quality (contrast-to-noise/signal-to-noise ratio) and whole-brain segmentation, subcortical limbic-specific QC (SM 1.5, Table S2) identified group-wise outliers based on (1) *z*-score deviations from FreeSurfer reference values [40], (2) segmentation confidence [40], (3) volume, and (4) bilateral symmetry (cut-off >2.698*SD [64, 65]). Scans with ≥1 outlier(s) underwent detailed visual inspection (FreeView [63]) of all subcortical limbic structures in all planes by a trained rater. Scans with significant artifacts were excluded after expert review (overall exclusion rate = 3.46%; no group differences; Table S3).

### Statistical analyses

Analyses (SM 1.6) were conducted in R v4.4.1 (R Core Team, 2024). All variables, including subcortical limbic volumes (Fig. S1), were approximately normally distributed. Group differences in demographics, clinical variables, marker concentrations, head size, and global gray matter were tested using general linear models (GLMs) and linear mixed effects models (LMEs) for single and repeated measures, respectively (Table 1).

All analyzed structures are adjacent to ventricles (Fig. 1). Given robust evidence for gray matter loss and compensatory ventricular enlargement in acute AN (Table S4) [2, 3], which may bias segmentation [66, 67], we assessed reliability via associations between subcortical limbic and adjacent ventricle volumes (Table S5). Expected inverse relationships were not consistently observed; instead, mammillary bodies and septal nuclei showed strong positive ventricle relationships (Table S5), indicating potential bias and leading to exclusion (SM 1.6). This was supported by discrepancies when comparing models with vs. without ventricle adjustment (Fig. S2).

**Fig. 2:**
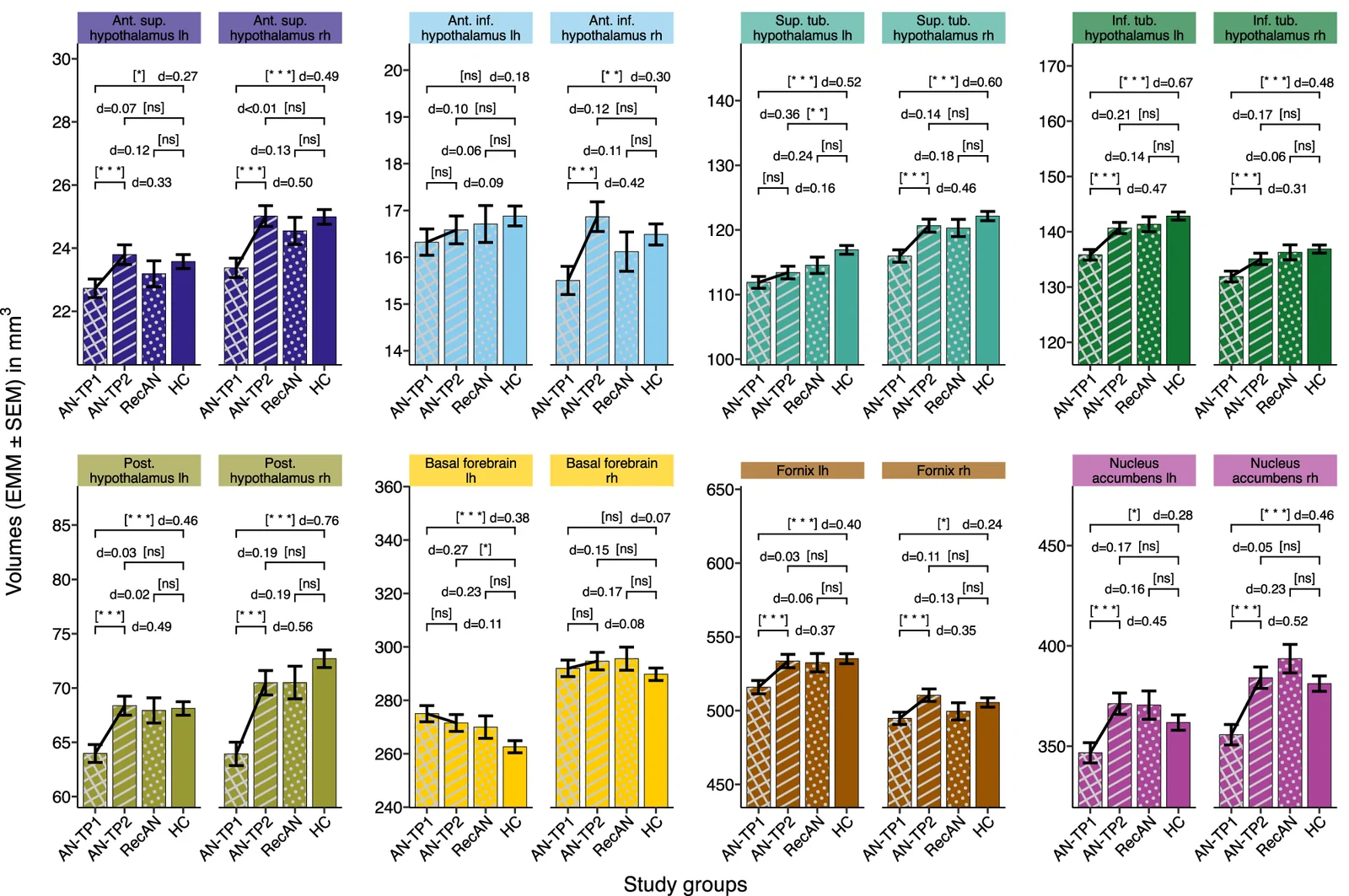
Cross-sectional and longitudinal group differences in subcortical limbic structures. AN participants with acute anorexia nervosa in the acutely underweight state (TP1) and reassessed after short-term weight-restoration (TP2), ant. anterior, HC healthy control participants, inf. inferior, lh left hemisphere, post. posterior, RecAN long-term weight-recovered participants with a history of AN, rh right hemisphere, sup. superior, tub. tubular. Main linear mixed effects (LME) model for group differences in subcortical limbic structures; after excluding bilateral mammillary bodies and septal nuclei due to potential segmentation bias. Cross-sectional group effects (AN, RecAN, HC) and longitudinal change AN-TP2-AN-TP1 (as a linear function of BMI-SDS increase) were modeled, covarying for linear and quadratic age effects and head size (please consult SM 1.6 for details). Bar plots display LME model estimates: estimated marginal mean (EMM) ± standard error of the mean (SEM) of individual volumes in each study group. FDR-adjusted significance (FDR-adjustment across all contrasts and all volumes) and effect size estimate Cohen’s *d* are presented for contrasts of interest (AN-TP2 vs. AN-TP1, AN-TP1 vs. HC, AN-TP2 vs. HC, and RecAN vs. HC). Significance levels: [***], FDR-*q* < 0.001; [**], FDR-*q* < 0.01; [*], FDR-*q* < 0.05; [ns], nonsignificant.

Cross-sectional (AN-TP1/TP2, RecAN, HC) and longitudinal (AN-TP2 vs. AN-TP1) differences in subcortical limbic volumes were analyzed using random-intercept LMEs adapted from our prior work [5, 6, 68]. Weight-restoration-related volumetric change was modeled as a function of BMI-SDS increase. Models included age effects (linear, quadratic) and head size as covariates (Fig. 2). False discovery rate (FDR)-adjustment [69] was applied across all contrasts and structures. Sensitivity analyses accounted for cigarette smoking, co-existing mental conditions, and antidepressant medication (Table 1 and Fig. S3). Bayesian LMEs assessed evidence for null effects (Table S6).

**Fig. 3:**
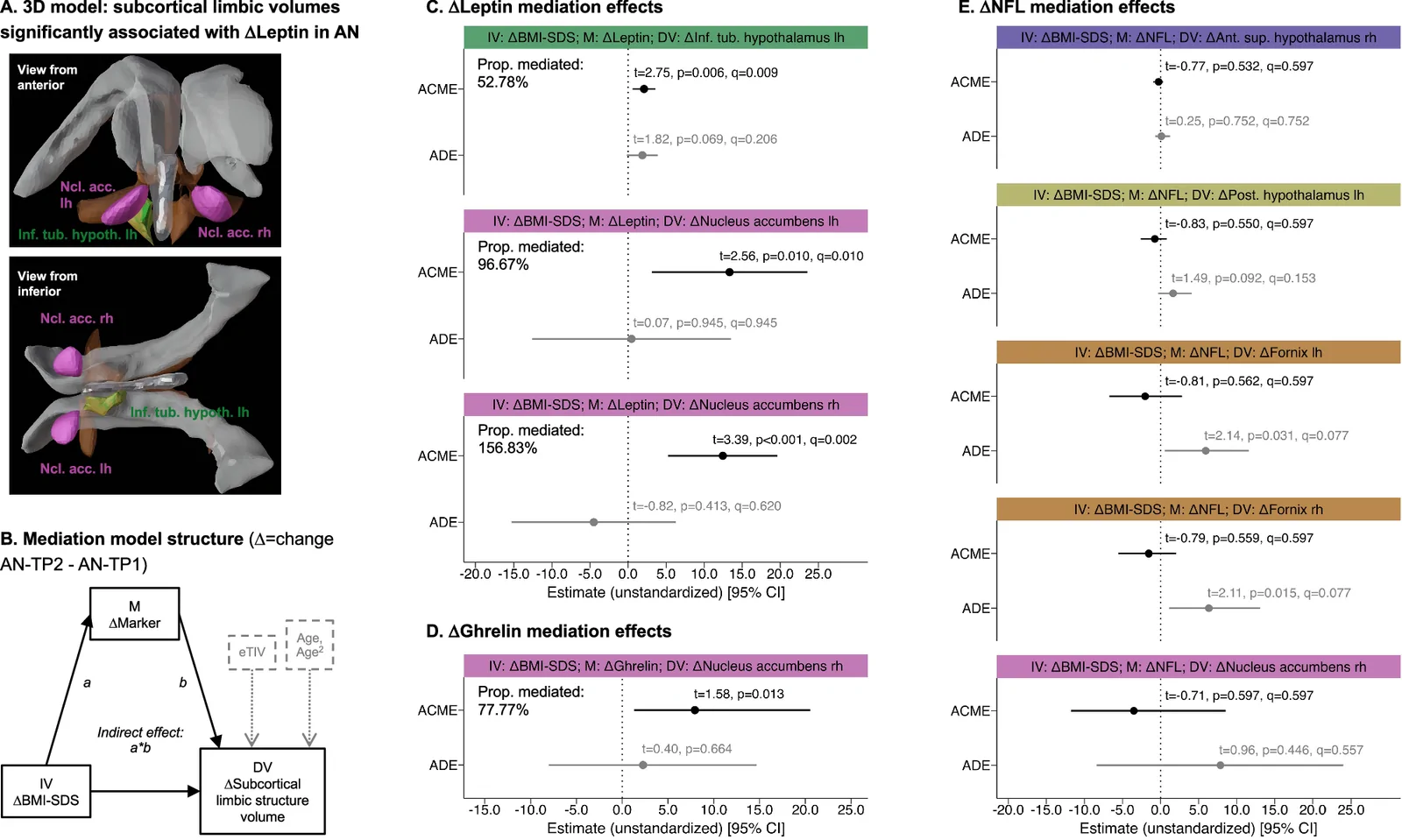
Mediation analysis in AN participants for longitudinal change in subcortical limbic structures following short-term weight-restoration, mediated by change in blood-based protein marker levels. AN participants with acute anorexia nervosa in the acutely underweight state (TP1) and reassessed after short-term weight-restoration (TP2), ant. anterior, BMI-SDS body mass index-standard deviation score, eTIV estimated total intracranial volume, hypoth. hypothalamus, inf. inferior, lh left hemisphere, NFL neurofilament light, ncl. acc. nucleus accumbens, post. posterior, rh right hemisphere, sup. superior, tub. tubular. **A** 3D model (obtained from FreeSurfer-processed T1-weighted MRI scan of a study participant [63, 108]) visualizing the anatomical location of those subcortical limbic structures that were significantly associated with leptin change scores following short-term weight-restoration and underwent subsequent mediation analysis. **B** Mediation model structure (in AN participants): weight gain (BMI-SDS increase) served as the independent variable (IV), change in marker levels (leptin, ghrelin, and NFL) as the mediator (M), and change in subcortical limbic structures, adjusted for linear and quadratic age effects and head size, as the dependent variable (DV). Multiple regression-based mediation analysis, mediation/indirect effect was computed as the product of coefficients of *path a* and *path b* [70]. No mediation model was established for BDNF due to the absence of longitudinal associations with subcortical limbic structures (Table 2D). Mediation analysis in AN participants for change in leptin levels (**C**), ghrelin levels (**D**), and NFL levels (**E**) following short-term weight-restoration and selected subcortical limbic structures, which were significantly associated with those marker levels (Table 2A–C in bold). Average (causal) mediation effects (ACME, through marker change scores) and average direct effects (ADE, corresponding to effects of weight gain on subcortical limbic dynamics after removing marker effects) are reported; unstandardized estimates (points) and their 95% confidence intervals (CI, horizontal lines) are presented. To obtain robust CIs, nonparametric bootstrapping using the percentile method with 10,000 simulations was performed. Further, *t*-value, *p* value, and FDR-q (FDR-adjustment across all mediation models per marker) are stated for ACME and ADE. Proportion (Prop.) mediated $\left(\frac{{{{\rm{ACME}}}}}{{{{\rm{ACME}}}}+{{{\rm{ADE}}}}}\right)$ was computed as effect size measure when significant mediation occurred.

For structures showing longitudinal change in AN (Fig. 2), associations with marker changes (leptin, ghrelin, NFL, BDNF; Fig. S4) beyond ΔBMI-SDS were tested using modified LMEs (Table 2). FDR-adjustment was applied across all markers and structures. Significant marker effects were followed by regression-based mediation analyses [70] (Fig. 3B). Supplementary LMEs tested longitudinal associations between subcortical limbic structures and additional demographic/clinical variables (age group, DOI, AN-subtype, and changes in ED and depressive symptoms; Table S7).

**Table 2:** Linear mixed effects models for longitudinal associations between change in blood-based protein marker levels and subcortical limbic structures following short-term weight-restoration in AN.

Subcortical limbic structures showing significant volumetric increase in AN at TP2 vs. TP1	LME model: Subcortical limbic structure volume = Intercept_Random_ + Δ_AN_ + Δ_RecAN_ + A_AN_*(BMI-SDS_TP(x)_ − BMI-SDS_TP1_) + B_AN_*(Marker_TP(x)_ − Marker_TP1_) + C*Age + CC*Age^2⊥^ + D*eTIV																							
	Endocrine markers												Neuronal markers											
	A. ∆Leptin						B. ∆Ghrelin						C. ∆NFL						D. ∆BDNF					
	*β*	se	*t*	*p*	*q*	*d*	*β*	se	*t*	*p*	*q*	*d*	*β*	se	*t*	*p*	*q*	*d*	*β*	se	*t*	*p*	*q*	*d*
Ant. sup. hypothalamus lh	0.16	0.15	1.05	0.294	0.522	0.09	−0.02	0.01	−2.33	0.023	0.100	0.56	−0.57	0.58	−0.98	0.329	0.563	0.20	2.04	1.04	1.96	0.053	0.191	0.44
Ant. sup. hypothalamus rh	−0.10	0.17	−0.62	0.537	0.653	0.05	−0.02	0.01	−1.73	0.087	0.231	0.36	**−2.19**	**0.62**	**−3.52**	**<0.001**	**0.005**	**0.66**	1.70	1.15	1.48	0.142	0.344	0.29
Ant. inf. hypothalamus rh	−0.10	0.16	−0.61	0.544	0.653	0.05	−0.01	0.01	−1.08	0.283	0.522	0.21	−0.42	0.65	−0.65	0.519	0.653	0.12	0.80	1.18	0.68	0.499	0.653	0.13
Sup. tub. hypothalamus rh	0.40	0.49	0.81	0.416	0.595	0.07	−0.03	0.03	−1.20	0.234	0.450	0.27	−3.70	1.93	−1.92	0.058	0.191	0.38	0.51	3.45	0.15	0.884	0.903	0.03
Inf. tub. hypothalamus lh	**1.38**	**0.40**	**3.42**	**<0.001**	**0.005**	**0.28**	0.04	0.02	1.92	0.060	0.191	0.50	−0.35	1.56	−0.22	0.824	0.878	0.05	2.71	2.97	0.91	0.365	0.565	0.21
Inf. tub. hypothalamus rh	0.73	0.41	1.77	0.077	0.217	0.14	0.00	0.02	−0.10	0.920	0.920	0.02	0.33	1.67	0.20	0.842	0.878	0.04	1.80	3.06	0.59	0.558	0.653	0.13
Post. hypothalamus lh	0.41	0.44	0.95	0.343	0.565	0.08	−0.03	0.02	−1.24	0.217	0.435	0.29	**−4.86**	**1.69**	**−2.87**	**0.005**	**0.035**	**0.56**	−4.23	3.19	−1.32	0.188	0.393	0.27
Post. hypothalamus rh	0.15	0.52	0.28	0.780	0.851	0.02	−0.01	0.03	−0.49	0.623	0.695	0.11	−4.79	2.12	−2.26	0.026	0.103	0.44	−6.99	3.78	−1.85	0.068	0.203	0.39
Fornix lh	2.67	1.16	2.30	0.022	0.100	0.19	−0.03	0.06	−0.52	0.604	0.690	0.15	**−16.32**	**4.03**	**−4.05**	**<0.001**	**0.002**	**0.98**	−11.93	8.04	−1.48	0.143	0.344	0.39
Fornix rh	1.03	1.24	0.83	0.407	0.595	0.07	−0.05	0.07	−0.72	0.476	0.653	0.20	**−13.15**	**4.75**	**−2.77**	**0.007**	**0.043**	**0.66**	7.18	8.86	0.81	0.421	0.595	0.21
Nucleus accumbens lh	**10.50**	**2.51**	**4.18**	**<0.001**	**0.002**	**0.34**	0.08	0.13	0.61	0.541	0.653	0.15	−12.89	9.56	−1.35	0.181	0.393	0.27	−16.23	17.54	−0.93	0.357	0.565	0.20
Nucleus accumbens rh^a^	**9.13**	**2.36**	**3.87**	**<0.001**	**0.002**	**0.32**	**−0.46**	**0.13**	**−3.64**	**<0.001**	**0.005**	**0.91**	**−25.21**	**9.44**	**−2.67**	**0.009**	**0.048**	**0.56**	−25.08	17.95	−1.40	0.166	0.380	0.31

Modifications of the main LME model (SM 1.6, Fig. 2) to investigate longitudinal associations of change scores in leptin (log_e_-transformed; A.), ghrelin (power[¾]-transformed and adjusted for batch effects; B.), NFL (log_e_-transformed and adjusted for batch effects; C.), and BDNF (log_e_-transformed and adjusted for batch and storage time effects; D.) with subcortical limbic structures following short-term weight-restoration in AN (only those volumes with significant change AN-TP2-AN-TP1 at FDR-*q* < 0.050 according to the main LME model (Fig. 2) were analyzed; models included all study groups). Unstandardized coefficient (*β*), its standard error (se), *t*-value, unadjusted *p* value (two-tailed), FDR-*q* (FDR-adjustment across all volumes and all markers), and Cohen’s *d* are stated. Significant associations are highlighted in bold.

*AN* participants with acute anorexia nervosa in the acutely underweight state (TP1) and reassessed after short-term weight-restoration (TP2), *ant.* anterior, *BDNF* brain-derived neurotrophic factor, *BMI-SDS* body mass index-standard deviation score, *eTIV* estimated total intracranial volume, *HC* healthy control participants, *inf.* inferior, *lh* left hemisphere, *NFL* neurofilament light, *post.* posterior, *RecAN* long-term weight-recovered participants with a history of AN, *rh* right hemisphere, *sup.* superior, *tub.* tubular.

^a^Right nucleus accumbens volumetric increase was significantly associated with changes in leptin, ghrelin, and NFL levels. Given a significant negative correlation between leptin and NFL in AN (Fig. S4), we developed an exploratory LME model incorporating ∆Leptin and ∆NFL main effects as well as their interaction term (covarying for ∆BMI-SDS, linear and quadratic age effects, and head size): ∆Leptin main effect (*β *= 12.01, se = 4.22, *t* = 2.85, *p* = 0.005, *d* = 0.24) and ∆NFL main effect (*β *= −58.69, se = 22.33, *t* = −2.63, *p* = 0.009, *d* = 0.22) were significant, while the ∆Leptin × ∆NFL interaction effect (*β *= 9.86, se = 5.53, *t* = 1.78, *p* = 0.075, *d* = 0.15) was nonsignificant.

## Results

### Descriptive statistics

Groups differed in age but not IQ, socioeconomic status, or handedness (Table 1A). Head size did not differ (Table 1C), consistent with our previous studies [5, 6]. BMI(-SDS) increased in AN but did not fully normalize following weight-restoration (duration: mean ± SD = 89 ± 27 days, range 35–224 days; ΔBMI: 29 ± 10%, 14–63%). ED-specific and depressive symptoms decreased but remained elevated in AN-TP2 vs. HC. RecAN showed slightly lower BMI-SDS than HC and residual symptoms (Table 1A).

Leptin was reduced and ghrelin elevated in AN-TP1, with both normalizing after short-term weight-restoration. NFL was elevated in AN-TP1, decreased longitudinally, but remained above HC levels at AN-TP2. In RecAN, leptin, ghrelin, and NFL were normal. No group differences emerged for BDNF (Table 1B and Fig. S4).

Total (−7.01%) and subcortical (−4.44%) gray matter volumes were reduced in AN-TP1 vs. HC, then increased and normalized at TP2 (Table 1D). Correspondingly, lateral and third ventricles were enlarged in AN-TP1 and decreased during weight-restoration (Table S4).

### Subcortical limbic structural dynamics

As anticipated, all subcortical limbic structures—except left anterior inferior hypothalamus and bilateral basal forebrain—were significantly smaller in AN-TP1 vs. HC (Figs. 2 and 3). Effect sizes were generally medium (Cohen’s *d* mean[range] = 0.46[0.24–0.76]), with an average reduction of −5.28%. Largest reductions were observed in the right posterior hypothalamus (−12.05%), the right nucleus accumbens (−6.67%), and the right anterior superior hypothalamus (−6.46%). Left anterior inferior hypothalamus showed no group or longitudinal differences. Left basal forebrain was mildly increased in AN-TP1 (+4.72%) and AN-TP2 (+3.40%) vs. HC.

Most structures increased following short-term weight-restoration (e.g., right posterior hypothalamus +10.24%, right anterior inferior hypothalamus +8.80%, right/left nucleus accumbens +7.98%/+7.06%) and fully normalized by AN-TP2 (Fig. 2). Changes were predominantly of medium magnitude (*d* mean[range] = 0.44[0.30–0.56]; mean increase = +5.78%; Fig. 2). In contrast, the left superior tubular hypothalamus showed no longitudinal change and remained reduced in AN-TP2 vs. HC. All structures were fully normalized in RecAN (Fig. 2).

Group differences were robust to potential confounders, specifically cigarette smoking, mental comorbidities, and SSRI/mirtazapine use (Fig. S3). Bayesian analyses provided strong evidence for no differences between AN-TP2 and RecAN vs. HC in most structures (Bayes factors >10; Table S6), supporting frequentist findings of normalization after short-term and long-term recovery (Fig. 2).

### Marker associations with subcortical limbic dynamics

Increasing leptin levels in AN predicted increases in the left inferior tubular hypothalamus and bilateral nucleus accumbens, independent of ΔBMI-SDS (Table 2A and Fig. 3A). Leptin fully mediated the relationship between weight gain and volumetric increase in these structures (Fig. 3C). Declining ghrelin levels predicted right nucleus accumbens increase; they also fully mediated its weight gain-related change (Table 2B and Fig. 3D).

Decreasing NFL levels in AN predicted volumetric increases in five structures—right anterior superior hypothalamus, left posterior hypothalamus, bilateral fornix, and right nucleus accumbens—independent of ΔBMI-SDS, yielding medium-to-large effect sizes (*d* mean[range] = 0.69[0.56–0.98]; Table 2C). However, NFL did not mediate any weight gain-related structural dynamics (Fig. 3E).

Although mean BDNF was unchanged from AN-TP1 to AN-TP2 (Table 1B), individual BDNF change scores varied (Fig. S4). However, they did not predict subcortical limbic dynamics during weight-restoration (Table 2D).

Increases in right nucleus accumbens volume were significantly associated with longitudinal changes in leptin, ghrelin, and NFL (Table 2A–C). Leptin and NFL were significantly cross-correlated in AN (Fig. S4). Follow-up analysis revealed significant main effects of leptin and NFL change on right nucleus accumbens increase, with a nonsignificant interaction (Table 2, footnote a), indicating largely independent effects.

### Further demographic and clinical influences on subcortical limbic dynamics

Age group (adolescent vs. adult) did not affect the normalization rate of any subcortical limbic structure in AN (Table S7A). Furthermore, DOI and AN-subtype (restrictive vs. binge/purge) did not influence weight gain-related increases of subcortical limbic volumes (Table S7B, C). Likewise, reductions in ED and depressive symptoms were unrelated to structural changes during weight-restoration, beyond ΔBMI-SDS (Table S7D, E).

## Discussion

To our knowledge, this is the first large-scale study systematically examining alterations in subcortical limbic structures, including hypothalamic subregions, and their associations with endocrine and neuronal markers in girls and young women with AN, recovered individuals, and healthy controls, using a combined cross-sectional and longitudinal design across nutritional states. As hypothesized, most subcortical limbic structures showed volumetric reductions of overall medium effect size in acutely underweight patients, largely normalizing after short-term weight-restoration and remaining stable in individuals weight-recovered for an average of five years. This pattern aligns with prior findings on cortical and subcortical gray and white matter alterations in AN [2–6] and supports a state-dependent rather than trait-related interpretation [38]. Effects were robust to potential confounding by lifestyle (cigarette smoking) and clinical factors (mental comorbidities and psychoactive medication).

However, not all regions followed this pattern: the left anterior inferior hypothalamus remained unchanged, and the left basal forebrain was enlarged in both underweight and short-term weight-restored states, suggesting relative resilience to weight-related changes in AN. In contrast, the left superior tubular hypothalamus remained reduced after short-term—but not long-term—weight-restoration, indicating incomplete short-term recovery and suggesting potentially associated functional impairment that may persist beyond discharge from acute treatment.

Leptin, ghrelin, and NFL levels changed as expected following short-term weight-restoration [16, 22, 31]. Cross-sectional and longitudinal BDNF differences were nonsignificant despite individual dynamics in AN, consistent with some but not all prior findings [26, 34–37, 61]. Increases in leptin and decreases in ghrelin independently mediated weight gain-related volumetric increases in the left inferior tubular hypothalamus (leptin only) and the nucleus accumbens (left: leptin only; right: leptin and ghrelin). Decreasing NFL levels were associated with volumetric normalization in two hypothalamic subunits (right anterior superior and left posterior hypothalamus), the bilateral fornix, and the right nucleus accumbens, independent of the amount of weight gain. Despite being cross-correlated, leptin and NFL both showed significant main effects on right nucleus accumbens increase, with no evidence of interaction, indicating additive, independent contributions. These findings highlight potential prognostic and/or therapeutic relevance of leptin, ghrelin, and NFL for subcortical limbic recovery over the course of short-term weight-restoration in AN.

The magnitude of structural alterations in AN and their responsiveness to weight gain varied across regions, with effect sizes ranging from small (e.g., basal forebrain) to medium or large (e.g., inferior tubular and posterior hypothalamus, nucleus accumbens). Overall, alterations in AN (averaged *d* = 0.46) exceeded those reported in other psychiatric disorders. In schizophrenia, only small effects have been observed for hypothalamic subunits (*d* < 0.24) [71] and the nucleus accumbens (*d* = 0.25) [72]. Similarly, changes in AN surpassed those reported in major depressive disorder for the nucleus accumbens (*d* = 0.02) [73] and hypothalamus (*d* = 0.39) [74].

Using a larger sample and hemisphere-specific analyses, we replicated and extended findings from a prior cross-sectional study, reporting reduced inferior tubular hypothalamus volume, averaged across hemispheres, in acute AN (*n* = 78) [49]. We identified additional hypothalamic subunits affected by AN, including bilateral superior tubular and posterior hypothalamus. This discrepancy likely reflects lower statistical power, higher mean age ( ≈ 23 vs. ≈16 years), longer illness duration ( ≈ 65 vs. 14 months), and greater SSRI exposure ( ≈ 40% vs. 2%) in the previous study vs. the current study [49]. The only other study reported total hypothalamic enlargement in AN but included only 13 patients, was cross-sectional, and used a different segmentation approach [50], limiting statistical power and comparability. However, a methodological strength is its use of 7 T imaging [50].

Prolonged undernutrition and altered neurohormonal and neurotransmitter systems have led to hypotheses of mitochondrial dysfunction and hypothalamic neurodegeneration in AN [75], analogous to classical neurodegenerative disorders [76]. However, this interpretation is not supported by our data, as most hypothalamic alterations were reversible following short-term weight-restoration. The exception was the left superior tubular hypothalamus, which remained reduced at AN-TP2 follow-up. This region, comprising the lateral hypothalamus, dorsomedial nucleus, and paraventricular nucleus, regulates energy balance and feeding motivation [41, 77]. Persistent reductions may therefore relate to residual ED symptoms after short-term weight-restoration.

Conversely, the left anterior inferior hypothalamus and left basal forebrain exhibited relatively limited volumetric dynamics across nutritional states of AN. The anterior inferior hypothalamus contains the suprachiasmatic and supraoptic nuclei, which regulate circadian rhythms, oxytocin secretion, and water balance through vasopressin [41, 77]. Accordingly, endogenous oxytocin and vasopressin metabolism seems relatively preserved in AN [78, 79]. The slight enlargement of the basal forebrain in acute and short-term weight-restored AN—an area involved in arousal, attention, and learning [80]—may reflect impaired cognitive flexibility reported in patients with AN before and after short-term weight-restoration [81, 82].

Although prior smaller-scale cross-sectional studies (*n* = 23 [49]; *n* = 13 [50]) did not detect associations between hypothalamic volume and leptin in acute AN, our longitudinal findings in a larger AN cohort undergoing weight-restoration show leptin-mediated increases in left inferior tubular hypothalamus volume. This region includes the arcuate nucleus [41, 77], which has the brain’s highest leptin receptor density [42], facilitates central leptin signaling [83], stimulates leptin-induced hypothalamic neurogenesis [84], and initiates food intake [45]. Thus, by acting on the arcuate nucleus, increasing leptin during weight-restoration may enhance neuroplasticity and support structural recovery. We also observed left-lateralized leptin effects. Consistent with this, limited evidence suggests left hypothalamic dominance in feeding behavior and metabolic homeostasis in fasting rodents [85].

In addition, we demonstrated mediation effects of leptin on volumetric normalization of the bilateral nucleus accumbens, a key component of the dopaminergic ventral striatum that integrates reward-related signaling and interacts with hypothalamic and limbic circuits [7, 10, 26, 47]. Extending prior findings regarding leptin effects on amygdala subnuclei [5], leptin may also contribute to nucleus accumbens structural recovery and thereby alleviate affective symptoms associated with its alterations in acute AN [9–11]. Case reports of metreleptin treatment in patients with AN describe rapid improvements in ED-related cognitions, mood, and hyperactivity, enhancing treatment adherence and clinical outcomes [16, 86, 87]. A phase-2 randomized, placebo-controlled, quadruple-blind metreleptin trial is ongoing (NCT06305182).

Moreover, declining ghrelin levels mediated normalization of the right nucleus accumbens, where ghrelin promotes dopamine release and food-related reward [10, 26, 46]. This link may be understood against the background of AN-related ghrelin resistance despite elevated ghrelin secretion [15, 22]. Endocrine rehabilitation, reflected by decreasing peripheral ghrelin [22], may restore central ghrelin sensitivity and enable its neurogenic effects [88], suggesting a potential role in neurostructural recovery. Treatment with the partial ghrelin receptor agonist relamorelin in AN improved gastric emptying, accompanied by a trend-level increase in weight [89].

In addition to the right nucleus accumbens and two hypothalamic subunits involved in cognition and feeding (e.g., posterior hypothalamus) [41, 77], decreasing NFL levels in AN were independently associated with volumetric normalization of the bilateral fornix—a white matter bundle connecting limbic system structures [40, 90]. This longitudinal NFL-fornix association may reflect attenuation of axonal damage, likely present in acute AN [28, 30, 31], and restitution of neurostructural integrity. In support, NFL is recognized as a neuronal injury marker across neurological and psychiatric disorders (e.g., multiple sclerosis, psychosis, depression) [29]. Correspondingly, elevated NFL levels have been linked to focal cortical thinning and white matter volume reductions in AN [32, 33]. Notably, this study provides the first longitudinal evidence connecting NFL with subcortical limbic dynamics across nutritional states of AN.

In contrast, BDNF was not associated with subcortical limbic changes in AN, differing from prior evidence of hippocampal effects [32]. Aligning with the literature [91], BDNF may exert more pronounced effects on hippocampal plasticity than on other limbic structures examined here.

Collectively, the identified links of leptin, ghrelin, and NFL with limbic dynamics offer novel insights into potential mechanisms underlying the reversibility of neurostructural alterations in AN. Additionally, these markers can be measured in peripheral blood in a minimally invasive, reliable, and cost-effective manner. Given the high standardized mortality rate of AN among mental disorders [1, 92] and the lack of targeted pharmacotherapy [38], revealing clinically relevant neurobiological markers is particularly important.

Study strengths include a large sample, a predominantly psychopharmacologically naïve cohort, standardized MRI and biomarker assessment protocols, and rigorous QC procedures. Structures were included only after careful evaluation for segmentation bias related to AN-associated tissue loss and ventricular enlargement. Although the reliability of fornix segmentation has been questioned [67], our findings were hypothesis-consistent and showed no evidence of bias in QC or sensitivity analyses. Mammillary bodies and septal nuclei were excluded due to concerns about segmentation accuracy.

Several limitations warrant consideration. First, the sample comprised young, nonchronic female AN participants of European descent, limiting generalizability to other populations. Second, binge/purge AN-subtype was underrepresented but showed negligible effects on subcortical limbic dynamics. Third, more frequent assessments in AN during weight-restoration and longitudinal assessment of HC could further clarify trajectories. Fourth, the relatively smaller BDNF sample (cf. Table 1) may have limited statistical power to detect neurostructural associations. Fifth, although markers were assessed peripherally, given the limited feasibility of cerebrospinal fluid (CSF) collection in humans, substantial evidence supports their role as indirect indicators of central processes: leptin and ghrelin cross the blood-brain barrier, correlate with CSF levels, and influence neural circuits [93–96]; BDNF also shows blood-brain barrier passage and central-peripheral correspondence [97, 98]; and NFL is released following central axonal injury with strong cross-compartment correlations [99, 100]. Finally, markers were selected based on strong evidence for neurostructural associations [5, 6, 19, 25, 32, 33, 37] and established roles in limbic circuits [10, 26, 27]. Future research should extend this framework to additional signals, such as proteomic findings [101–103].

## Conclusion

This study demonstrates pronounced neuroplasticity in subcortical limbic structures across nutritional states in AN. Changes in leptin, ghrelin, and NFL predict and/or mediate structural dynamics in key reward and homeostatic regions, particularly the nucleus accumbens and hypothalamic subcenters. Consequently, the reversibility of subcortical limbic alterations following weight-restoration may be partly explained by neuroendocrine recovery and the cessation of axonal damage. Further large-scale and interventional studies are needed to evaluate leptin-based treatment in AN. Modulating ghrelin signaling represents another promising approach, while NFL may indicate structural changes in the limbic system. Ultimately, these advances may enable more precise diagnostic, prognostic, and therapeutic strategies for AN [38].

## Supplementary information

Supplementary Material to: Wronski et al.: Nutritional state-dependent changes in subcortical limbic structures in anorexia nervosa are linked to leptin, ghrelin, and neurofilament light

## Data Availability

The data that support the findings of this study contain confidential clinical information and are therefore not publicly available. De-identified data may be made available from the corresponding author upon reasonable request, subject to applicable ethical approvals and data protection regulations.
