# Supplementary material for: Nutritional state-dependent changes in subcortical limbic structures in anorexia nervosa are linked to leptin, ghrelin, and neurofilament light
Source: Neuropsychopharmacology. 2026 May 21;51(11):1932–45. doi: 10.1038/s41386-026-02435-w (PMC13597489; doi:10.1038/s41386-026-02435-w)
Supplement: Supplementary file 1 [file 41386_2026_2435_MOESM1_ESM.pdf]

Supplementary Material to:

Wronski et al.: Nutritional state-dependent changes in subcortical limbic structures in anorexia nervosa are linked to leptin, ghrelin, and neurofilament light

SM 1 Supplementary methods

SM 1.1 Participants

Additional exclusion criteria applied to all study participants (AN-TP1 and AN-TP2, participants with acute anorexia nervosa [AN]; RecAN, long-term weight-recovered participants with a history of AN; HC, healthy control participants), most importantly:

- Consumption of any psychoactive pharmakon within four weeks prior to the study (except for selective serotonin reuptake inhibitors [SSRIs] and mirtazapine in AN and RecAN participants; SSRIs were taken by 3 AN-TP1, 2 AN-TP2, and 4 RecAN; mirtazapine by 1 AN-TP1, 1 AN-TP2, and 1 RecAN),
- Substance abuse/dependence,
- Any history of organic brain syndrome, schizophrenia, bipolar disorder, or psychosis not otherwise specified,
- Current inflammatory, neurologic, or metabolic disorders,
- (Head) trauma and/or concussion,
- Chronic illness that could affect appetite, eating behavior, or body weight,
- Pregnancy or breastfeeding,
- Anemia,
- IQ below 85,
- MRI contraindications (e.g., medically or psychologically unstable patients with AN, ferromagnetic implants, orthodontic braces, large tattoos).

Further, current or former underweight was excluded in HC (i.e., current BMI <10th age percentile [for participants aged <18 years] or <18.5kg/m^2 [for participants aged ≥18 years], minimal lifetime BMI <10th age percentile [for participants aged <18 years] or <17.5kg/m^2 [for participants aged ≥18 years]). In both RecAN and HC, obesity was excluded (i.e., BMI ≥95th age percentile [for participants aged <18 years] or ≥30kg/m^2 [for participants aged ≥18 years]).

Table S1.1. Baseline (TP1) comparison between AN subgroups with both TP1 and TP2 assessments and those with only TP1 assessment

Characteristic	AN-TP1 with TP1 and TP2 assessments (n=113)	AN-TP1 with only TP1 assessment (n=55)	Independent samples t-test		
	Mean ± SD	Mean ± SD	t	p	d
Age (years)	16.11 ± 2.34	16.92 ± 4.10	-1.37	0.174	0.27
IQ	112.97 ± 12.26	108.82 ± 11.39	2.03	0.046	0.35
SES	3.40 ± 0.96	3.29 ± 0.85	0.69	0.490	0.11
BMI (kg/m^2)	14.77 ± 1.22	14.51 ± 1.73	1.04	0.303	0.19
BMI-SDS	-3.12 ± 1.04	-3.48 ± 1.57	1.52	0.133	0.29
EDI-2 core score	25.90 ± 7.11	26.11 ± 6.85	-0.19	0.853	0.03
BDI-II total score	23.54 ± 10.90	23.11 ± 11.60	0.23	0.821	0.04
Leptin (ng/mL)	1.55 ± 2.31	1.53 ± 2.28	n/a	n/a	n/a
Leptin-log$_e$	-0.90 ± 2.18	-1.33 ± 2.52	1.03	0.305	0.19
eTIV (mm^3)	1,498,395 ± 125,044	1,483,849 ± 120,500	0.73	0.470	0.12

Abbreviations: AN, participants with acute anorexia nervosa in the acutely underweight state (TP1) and reassessed after short-term weight-restoration (TP2); BDI-II total, total score of Beck Depression Inventory-II; BMI(-SDS), body mass index(-standard deviation score); EDI-2 core, averaged score comprising the core subscales drive for thinness, body dissatisfaction, and bulimia of Eating Disorder Inventory-2; eTIV, estimated total intracranial volume; IQ, intelligence quotient; SES, socioeconomic status.

Mean value and standard deviation (SD) are stated for each AN subgroup and variable (leptin was log$_e$-transformed to approximate normality). Group differences were tested using independent samples t-tests. As test statistics, t-value, two-tailed p-value, and effect size estimate Cohen's d are reported.

SM 1.2 Psychiatric assessments

The expert form of the Structured Interview for Anorexia and Bulimia Nervosa (SIAB-EX)[1], a well-validated 87-item semi-standardized interview with good inter-rater reliability (range 0.81-0.85)[2], was applied to evaluate the presence and severity of lifetime (from onset of puberty) or current (within three months before the study) eating-related psychopathology including assessment of AN-subtype. SIAB-EX interviews were conducted by clinically experienced and trained research assistants under supervision of a child and adolescent psychiatrist. Co-existing psychiatric diagnoses other than eating disorders were derived according to standard practice from medical records and confirmed by a board-licensed child and adolescent psychiatrist with over ten years of clinical experience.

SM 1.3 Leptin, ghrelin, NFL, and BDNF measurements and processing

For leptin (plasma), ghrelin (total, plasma), NFL (serum), and BDNF (serum) measurements, fasting venous blood samples were obtained (EDTA tubes for leptin and ghrelin; serum separator tubes for NFL and BDNF) and immediately processed as follows: addition of the serine protease inhibitor aprotinin and instant centrifugation (ϑ=5°C; a=2,500*g; for 15min) for leptin and ghrelin vs. centrifugation after a coagulation period of 30min for NFL and BDNF; aliquotation; storage at ϑ=-80°C until laboratory analysis. Lower limit of detection (LOD) was 0.20ng/mL for the leptin ELISA, 156pg/mL for the ghrelin ELISA, 0.104pg/mL for the NFL SIMOA assay, and 0.02ng/mL for the BDNF ELISA.

Values below LOD did not occur for ghrelin, NFL, and BDNF. Leptin values below LOD occurred in n=39 AN-TP1. Leptin values below LOD were imputed using censored likelihood maximization-based multiple imputation (CLMI)[3]. Missing leptin values due to unavailable blood samples were not imputed. Our CLMI model included the following predictors based on analysis models and theoretical and/or correlational relationships with leptin in AN-TP1:

- Volumes of individual subcortical limbic structures,
- Linear and quadratic effects of participant age,
- Head size (eTIV),
- BMI-SDS,
- Minimal lifetime BMI,
- Duration of illness,
- EDI-2 core score (eating disorder symptoms),
- BDI-II total score (depressive symptoms),
- AN-subtype according to SIAB-EX,
- Physical activity according to SIAB-EX.

CLMI procedure:

- (1) Construct a conditional censored likelihood distribution function of logarithmically transformed leptin values (leptin-$\log_e$),
- (2) Draw 50 random samples for leptin ***(<LOD)*** from the fitted truncated distribution resulting in 50 imputed datasets per subcortical limbic structure (only differing in leptin values),
- (3) Fit the analysis (linear mixed effects) models separately on each imputed dataset,
- (4) Pool individual analysis model parameters according to Rubin's combination rules for overall statistical inference accounting for within-imputation variance as well as between-imputation variance[4].

SM 1.4 Effects of batch and storage time on blood-based protein marker concentrations

Leptin values were log$_e$-transformed prior to analysis to reduce deviations from normality. Batch and storage time did not affect leptin values (Table S1.4A). Thus, no residualization was applied. Ghrelin values were power(¾)-transformed *($x^{0.75}$)* to reduce deviations from normality (mild positive skewness). Batch but not storage time affected ghrelin values (Table S1.4B). Thus, ghrelin values were residualized to account for batch effects prior to analysis (simplified formula: ghrelin$^{0.75}$ ~ ghrelin batch). NFL values were log$_e$-transformed to reduce deviations from normality. Batch but not storage time affected NFL values (Table S1.4C). Thus, NFL values were residualized to account for batch effects prior to analysis (simplified formula: NFL-log$_e$ ~ NFL batch). BDNF values were log$_e$-transformed to reduce deviations from normality. Batch and storage time affected BDNF values (Table S1.4D). Thus, BDNF values were residualized to account for batch effects and storage time prior to analysis (simplified formula: BDNF-log$_e$ ~ BDNF batch + storage time).

Table S1.4. Blood-based protein markers: batches and storage time

Marker	Batch		Storage time	
	$n_{Samples}$ per batch	*Linear model, adjusted for study group: t; p*	*Mean ± SD (days)*	*Linear model: t; p*
**A. Leptin**	Batch A[a]: 115	/	783.55 ± 504.55	0.91; 0.366
	Batch B[a]: 144	1.34; 0.182		
	Batch C[a]: 110	-1.51; 0.132		
	Batch D[a]: 222	0.63; 0.527		
**B. Ghrelin**	Batch A[b]: 69	/	2,834.94 ± 1,011.75	-0.36; 0.716
	Batch B[b]: 143	-3.12; 0.002		
	Batch C[b]: 231	-1.74; 0.082		
**C. NFL**	Batch A[b]: 100	/	1,889.75 ± 984.88	-1.13; 0.259
	Batch B[b]: 126	5.01; <0.001		
	Batch C[b]: 46	3.61; <0.001		
	Batch D[b]: 118	0.45; 0.652		
	Batch E[b]: 124	2.82; 0.005		
	Batch F[b]: 36	-1.89; 0.059		
**D. BDNF**	Batch A[b]: 113	/	725.80 ± 302.04	-2.99; 0.003
	Batch B[a]: 153	1.73; 0.084		
	Batch C[a]: 93	4.42; <0.001		

Abbreviations: BDNF, brain-derived neurotrophic factor; NFL, neurofilament light.

[a] Single measurement of marker concentration. [b] Duplicate measurements of marker concentration (mean value of both measurements was analyzed).

SM 1.5 Quality control of structural MRI scans

Table S2. MRI quality control procedure

**1. General whole-brain structural MRI QC**	• *Overall scan quality check:* a-priori exclusion of scans with low contrast-to-noise ratio/signal-to-noise ratio (CNR/SNR < mean - 2.698*SD)<br>• *General cortical and subcortical QC:* following standardized procedures of the ENIGMA consortium (http://enigma.ini.usc.edu/), including visual inspection of HTML-snapshots of brain surfaces and volumes of all scans and detailed visual inspection using FreeView of scans with statistical outliers and/or artifacts in HTML-snapshots
**2. Statistical outlier detection for subcortical limbic structures**	• Automated FreeSurfer quality assessment output (https://surfer.nmr.mgh.harvard.edu/fswiki/ScLimbic): z-score indicating deviation from FreeSurfer's reference standard and confidence as the mean posterior probability within a label (deep-learning neural network)<br>o Study group-wise high *z-score outliers*: > mean + 2.698*SD<br>o Study group-wise low *confidence outliers*: < mean - 2.698*SD<br>• Study group-wise *volume outliers* per structure and hemisphere: subcortical limbic volumes deviating more than mean ± 2.698*SD<br>• Study group-wise *bilateral symmetry outliers* per structure via lateralization index[5] (difference left-right hemisphere volumes divided by sum left+right hemisphere volumes): subcortical limbic structures deviating more than mean ± 2.698*SD
**3. Visual QC for subcortical limbic structures**	• Scans with any type of outlier (n≥1, either z-score, confidence, volume, or symmetry outlier(s)) in any subcortical limbic structure underwent detailed dynamic visual QC via FreeView of all subcortical limbic structures in all planes<br>• Visual QC was conducted by one trained rater using the T1-weighted image and colored subcortical limbic segmentation overlay in FreeView<br>• Suspicious cases were discussed in an expert meeting
**4. Subcortical limbic QC rating**	• Dichotomous rating for each scan: inclusion vs. exclusion<br>• Scans with artifacts in visual QC and/or segmentation failure were excluded prior to analysis (entire scan/all structures excluded)<br>• Common exclusion criteria were:<br>o Tissue misclassification (e.g., white matter inclusion),<br>o Unsegmented regions ("holes" in segmentation),<br>o Merging regions ("islands"), i.e., unclear separation of labels

Abbreviations: QC, quality control.

Outlier thresholds (mean ± 2.698*SD, corresponding to 1.5*interquartile range below first quartile or above third quartile) in accordance with ENIGMA guidelines for FreeSurfer-based (hippocampal) substructure segmentation[6,7].

Table S3. MRI quality control results

		**Scans** $n_{excl.}$ / n_{total}	$\%_{excl.}$	**Chi-squared test or** (if n<5) **Fisher's exact test:** p
**General whole-brain QC**				
Scan quality outliers (contrast-to-noise ratio, signal-to-noise ratio)	Overall	12 / 1031	1.16	
	AN-TP1	3 / 237	1.27	0.555
	AN-TP2	3 / 139	2.16	
	RecAN	1 / 204	0.49	
	HC	5 / 451	1.11	
General cortical and subcortical visual QC exclusions	Overall	37 / 1019	3.63	
	AN-TP1	10 / 234	4.27	0.159
	AN-TP2	9 / 136	6.62	
	RecAN	5 / 203	2.46	
	HC	13 / 446	2.91	
**Subcortical limbic QC**: n=227 of 982 scans with ≥1 outlier(s) of any type (Table S2) in any subcortical limbic structure underwent visual QC				
Subcortical limbic visual QC exclusions	Overall	34 / 982	3.46	
	AN-TP1	6 / 224	2.68	0.898
	AN-TP2	4 / 127	3.15	
	RecAN	7 / 198	3.54	
	HC	17 / 433	3.93	

Abbreviations: AN, participants with acute anorexia nervosa in the acutely underweight state (TP1) and reassessed after short-term weight-restoration (TP2); HC, healthy control participants; RecAN, long-term weight-recovered participants with a history of AN.

Number of excluded structural scans ($n_{excl.}$) and total number of available scans (n_{total}) are given for the overall sample and study group-wise (AN-TP1, AN-TP2, RecAN, HC) at each QC stage. **Post-QC sample consisted of 218 AN-TP1 scans, 123 AN-TP2 scans, 191 RecAN scans, and 416 HC scans** (all study groups: number of post-QC scans > number of participants). Please note that the main analysis sample was smaller, since the post-QC sample still included multiple scans of several participants who participated in two or more study arms (i.e., >1 scan/participant available). Subsequently, to generate the main analysis sample, only one scan per participant was chosen based on a predefined hierarchy (please note that the number of participants remained unchanged; AN-TP1: first AN episode and scan closest to treatment initiation to minimize early treatment effects [all MRI scans in AN-TP1 were acquired within 96h after treatment initiation] and QC [fewest outliers]; AN-TP2: scan closest to treatment termination/discharge and QC [fewest outliers]; RecAN: most recent scan/longer duration of recovery and QC [fewest outliers]; HC: first study participation and QC [fewest outliers]).

SM 1.6 Statistical analyses

1. Preliminaries

Background

- All analyzed subcortical limbic structures have anatomical border(s) with ventricles (cerebrospinal fluid spaces)
- Acute AN: ventricles enlarged; subcortical gray matter volumes reduced *(Table 1, Table S4)*

→Potential segmentation bias (e.g., ventricle space misclassified as subcortical structure)

Method

- General linear models for individual subcortical limbic structures, predicted by adjacent ventricle volume(s), adjusted for age, age^2, and eTIV *(Table S5)*

→Expected: negative association (i.e., larger ventricle volumes ~ smaller subcortical limbic volumes)

→ *Positive association of large effect size (Cohen's d≥0.8)* →

Subcortical limbic structures excluded due to suspected segmentation bias

- Mammillary bodies (lh, rh)
- Septal nuclei (lh, rh)

↓ *Subcortical limbic structures included in analyses (n=16 of 20)*

2.1 Main analyses: group differences

Descriptive statistics *(Table 1)*

- General linear models for IQ, SES, hand preference, minimal lifetime BMI, eTIV (n/a at AN-TP2)
- Linear mixed effects models for age, BMI(-SDS); EDI-2 core score, BDI-II total score; leptin ($\log_e$), ghrelin (total, $x^{0.75}$-transf.), NFL ($\log_e$), BDNF ($\log_e$); total and total subcortical gray matter volume

Main random-intercept linear mixed effects (LME) models for subcortical limbic structures *(Figure 2)*

- Model formula (incl. all study groups): Subcortical limbic volume = $\text{Intercept}_{\text{Random}} + \Delta_{AN} + \Delta_{RecAN} + A_{AN}*(\text{BMI-SDS}_{TP(x)} - \text{BMI-SDS}_{TP1}) + B*\text{Age} + BB*\text{Age}^{2\perp} + C*\text{eTIV}$
- Contrasts of Interest: AN-TP2 vs. AN-TP1, AN-TP1 vs. HC, AN-TP2 vs. HC, RecAN vs. HC
- False discovery rate (FDR, Benjamini-Hochberg) adjustment across all contrasts and all volumes

→

Exploratory analysis for all subcortical limbic structures *(Figure S2)*

- Comparison: main LME models (2.1) vs. modified LME models additionally adjusting for volumes of adjacent ventricles (covariance approach)

↓ *Subcortical limbic structures with significant longitudinal dynamics (AN-TP2 vs. AN-TP1 contrast) underwent further analyses (2.2)*

2.2 Main analyses: longitudinal associations with blood-based protein markers

Modified LME models for subcortical limbic structures predicted by leptin, ghrelin, NFL, and BDNF change scores over the course of short-term weight-restoration *(Table 2, Figure 3, Figure S4)*

- Model formula (incl. all study groups): Subcortical limbic volume = $\text{Intercept}_{\text{Random}} + \Delta_{AN} + \Delta_{RecAN} + A_{AN}*(\text{BMI-SDS}_{TP(x)} - \text{BMI-SDS}_{TP1}) + \mathbf{B_{AN}*(Marker_{TP(x)} - Marker_{TP1})} + C*\text{Age} + CC*\text{Age}^{2\perp} + D*\text{eTIV}$
- FDR adjustment across all volumes and all markers

→ Mediation models in AN to follow-up on significant longitudinal associations with markers *(Figure 3)*

- Independent variable: ΔBMI-SDS (Δ = change score AN-TP2 - AN-TP1)
- Mediator: ΔLeptin; ΔGhrelin; ΔNFL (separate models per mediator)
- Dependent variable: ΔSubcortical limbic volume, adjusted for age, age^2, and eTIV

↓ *Test robustness of group differences and investigate further demographic and clinical associations with subcortical limbic structures*

3. Supplementary analyses

- Modified LME models for subcortical limbic structures additionally adjusting for dichotomous **covariates**: cigarette smoking, presence of co-existing psychiatric conditions, and current antidepressant intake *(Figure S3)*
- **Bayesian-framework** LME models to follow-up on nonsignificant group differences from main LME models *(Table S6)*
- Modified LME models for longitudinal associations of subcortical limbic structures with **age group** (<18 years vs. ≥18 years)**, DOI, AN-subtype, and symptom scores** (EDI-2 core and BDI-II total) *(Table S7)*

Abbreviations: AN, participants with acute anorexia nervosa in the acutely underweight state (TP1) and reassessed after short-term weight-restoration (TP2); BDI-II, Beck Depression Inventory-II; BDNF, brain-derived neurotrophic factor; BMI(-SDS), body mass index(-standard deviation score); DOI, duration of illness; EDI-2, Eating Disorder Inventory-2; eTIV, estimated total intracranial volume; HC, healthy control participants; IQ, intelligence quotient; lh, left hemisphere; NFL, neurofilament light; RecAN, long-term weight-recovered participants with a history of AN; rh, right hemisphere; SES, socioeconomic status; TP(x), study time point in AN participants (either TP1 or TP2).

SM 1.7 CONSORT flow diagram

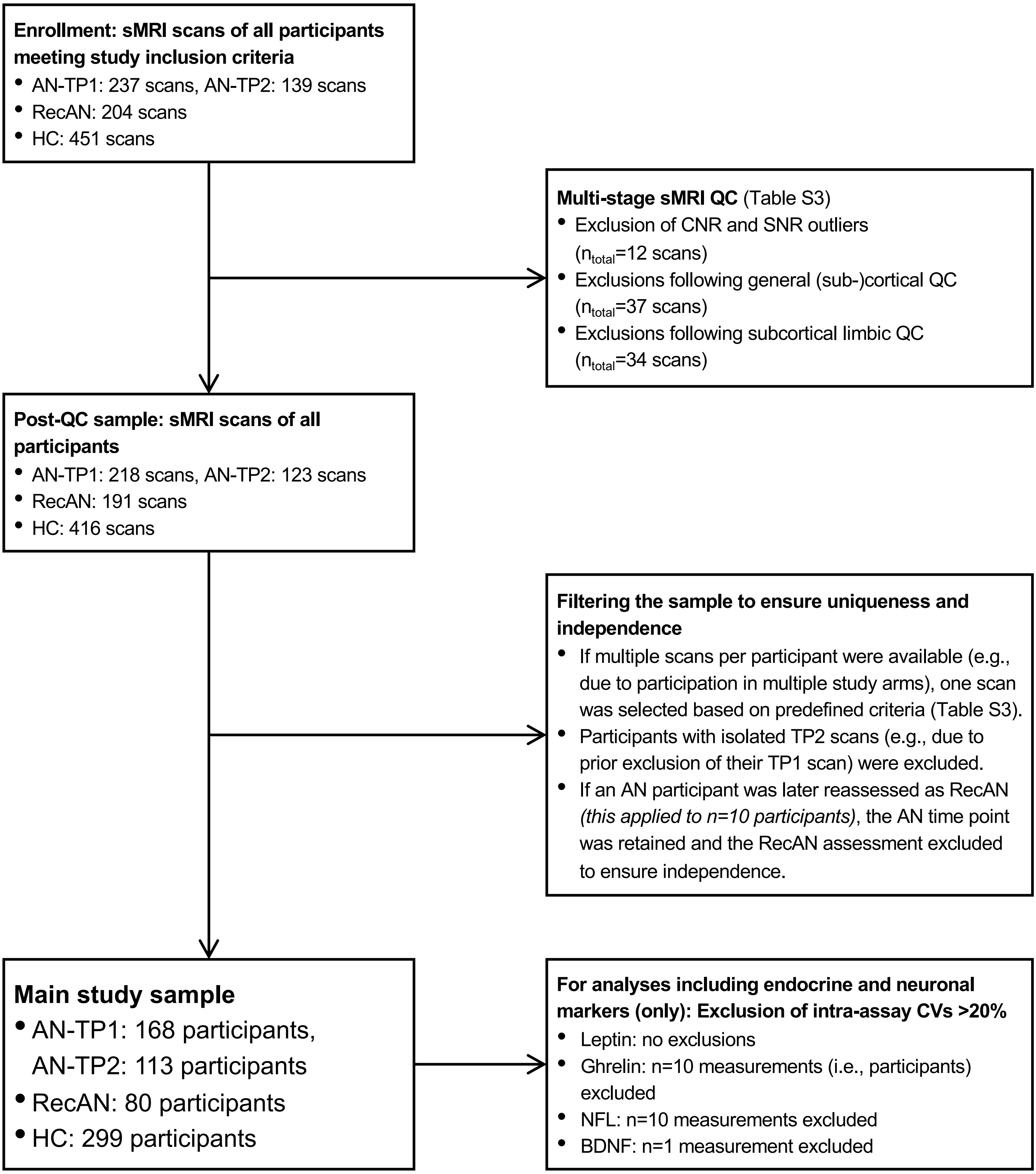

Abbreviations: AN, participants with acute anorexia nervosa in the acutely underweight state (TP1) and reassessed after short-term weight-restoration (TP2); BDNF, brain-derived neurotrophic factor; CNR, contrast-to-noise ratio; CV, coefficient of variation; HC, healthy control participants; NFL, neurofilament light; QC, quality control; RecAN, long-term weight-recovered participants with a history of AN; sMRI, structural MRI; SNR, signal-to-noise ratio.

SM 2 Supplementary results tables

Table S4. Ventricle volume group comparisons

**Ventricle**	**Acute AN group pre-/post treatment**				**Recovered group**		**Control group**		**Test statistics**			**Post-hoc contrasts**
	**AN-TP1**		**AN-TP2**		**RecAN**		**HC**					
	n	EMM ± SEM	n	EMM ± SEM	n	EMM ± SEM	n	EMM ± SEM	F (b/w | w/n)	q (b/w | w/n)	η_p^2 (b/w | w/n)	Significant at q<0.050
Lateral ventricle lh (mm³)	168	8,368 ± 273	113	6,605 ± 273	80	6,568 ± 379	299	5,957 ± 204	32.93 | 247.75	<0.001 | <0.001	0.11 | 0.66	AN-TP1>HC (q<0.001); AN-TP2<AN-TP1 (q<0.001); AN-TP2>HC (q=0.036)
Lateral ventricle rh (mm³)	168	7,511 ± 227	113	5,944 ± 228	80	6,011 ± 315	299	5,221 ± 170	43.36 | 275.62	<0.001 | <0.001	0.14 | 0.69	AN-TP1>HC (q<0.001); AN-TP2<AN-TP1 (q<0.001); AN-TP2>HC (q=0.006); RecAN>HC (q=0.020)
Third ventricle (mm³)	168	1,104 ± 18	113	936 ± 19	80	917 ± 25	299	861 ± 14	74.25 | 197.27	<0.001 | <0.001	0.21 | 0.59	AN-TP1>HC (q<0.001); AN-TP2<AN-TP1 (q<0.001); AN-TP2>HC (q<0.001); RecAN>HC (q=0.036)

Abbreviations: AN, participants with acute anorexia nervosa in the acutely underweight state (TP1) and reassessed after short-term weight-restoration (TP2); HC, healthy control participants; lh, left hemisphere; RecAN, long-term weight-recovered participants with a history of AN; rh, right hemisphere.

Ventricle volumes were extracted from FreeSurfer version 7.4 segmentation statistics[8]. Number of participants (n), covariate-adjusted estimated marginal mean (EMM), and standard error of EMM (SEM) are stated for each ventricle measure and study group. Group differences were tested using LME models, adjusted for age, age^2, and head size (eTIV). As test statistics, F-value (between-participants [b/w], i.e., AN-TP1-RecAN-HC | within-participant [w/n], i.e., AN-TP2-AN-TP1), FDR-q, and effect size estimate partial η^2 are reported. False discovery rate (FDR)[9] adjustment of p-values was applied across all ventricle measures and all contrasts of interest. Significant (q<0.050) post-hoc pairwise contrasts are stated.

Table S5. General linear models (study group-wise) for associations between volumes of subcortical limbic structures and anatomically adjacent ventricle volumes (adjusted for age, age^2, and eTIV)

**Subcortical (limbic) structure ~ adjacent ventricle**	**Study group: t; p; d**	
	**AN-TP1**	**AN-TP2**
Exploratory: total subcortical gray matter ~ lateral ventricle lh	*-3.42; <0.001; 0.54*	*-2.15; 0.034; 0.41*
Exploratory: total subcortical gray matter ~ lateral ventricle rh	*-4.34; <0.001; 0.68*	*-3.25; 0.002; 0.63*
Exploratory: total subcortical gray matter ~ third ventricle	*-2.08; 0.039; 0.33*	*-1.28; 0.203; 0.25*
Ant. sup. hypothalamus lh ~ third ventricle	0.10; 0.921; 0.02	1.72; 0.088; 0.33
Ant. sup. hypothalamus rh ~ third ventricle	-3.82; <0.001; 0.60	-1.06; 0.292; 0.20
Ant. inf. hypothalamus lh ~ third ventricle	0.72; 0.470; 0.11	0.22; 0.825; 0.04
Ant. inf. hypothalamus rh ~ third ventricle	-1.64; 0.103; 0.26	-2.66; 0.009; 0.51
Sup. tub. hypothalamus lh ~ third ventricle	-5.07; <0.001; 0.79	1.57; 0.119; 0.30
Sup. tub. hypothalamus rh ~ third ventricle	-3.06; 0.003; 0.48	0.82; 0.415; 0.16
Inf. tub. hypothalamus lh ~ third ventricle	-0.76; 0.451; 0.12	0.39; 0.701; 0.07
Inf. tub. hypothalamus rh ~ third ventricle	-2.29; 0.023; 0.36	-0.36; 0.717; 0.07
Post. hypothalamus lh ~ third ventricle	-0.64; 0.521; 0.10	-1.97; 0.052; 0.38
Basal forebrain lh ~ sum lateral (lh) and third ventricle	1.44; 0.153; 0.22	1.54; 0.127; 0.30
Basal forebrain rh ~ sum lateral (rh) and third ventricle	-1.93; 0.055; 0.30	-0.60; 0.547; 0.12
Fornix lh ~ sum lateral (lh) and third ventricle	-0.03; 0.978; <0.01	0.05; 0.959; <0.01
Fornix rh ~ sum lateral (rh) and third ventricle	-0.08; 0.932; 0.01	-0.71; 0.479; 0.14
Mammillary body lh ~ third ventricle[a]	*3.04; 0.003; 0.48*	1.15; 0.252; 0.22
Mammillary body rh ~ third ventricle	***5.87; <0.001; 0.92***	3.68; <0.001; 0.71
Nucleus accumbens lh ~ lateral ventricle lh	-2.81; 0.006; 0.44	-0.84; 0.404; 0.16
Nucleus accumbens rh ~ lateral ventricle rh	-3.40; <0.001; 0.53	-1.40; 0.163; 0.27
Septal nucleus lh ~ lateral ventricle lh	***6.92; <0.001; 1.08***	***5.77; <0.001; 1.11***
Septal nucleus rh ~ lateral ventricle rh	***6.23; <0.001; 0.98***	2.47; 0.015; 0.48

Abbreviations: AN, participants with acute anorexia nervosa in the acutely underweight state (TP1) and reassessed after short-term weight-restoration (TP2); ant., anterior; eTIV, estimated total intracranial volume; inf., inferior; lh, left hemisphere; post., posterior; rh, right hemisphere; sup., superior; tub., tubular.

All ventricle volumes were enlarged in AN participants at TP1 and TP2 (Table S4). **We expected negative associations between volumes of subcortical limbic structures and adjacent ventricle volumes, given overall head size (eTIV) remains constant, i.e., we anticipated smaller subcortical limbic structures in acute AN ~ larger ventricle volumes** (see *exploratory models for total subcortical gray matter volume* above for reference). Positive associations of subcortical limbic structures with adjacent ventricle volumes of large effect size (Cohen's d≥0.8) were considered indicative of potential segmentation bias. Thus, suspicious subcortical limbic structures, highlighted in ***bold-italics*** above, were excluded prior to main analyses (SM 1.6; [a]left-hemispheric mammillary body [highlighted in *italics*] was borderline but still excluded in accordance with right mammillary body).

Table S6. Bayesian LME model (Bayes factors) to follow-up on nonsignificant group differences from the main LME model

Subcortical limbic structure	BF_{01} for nonsignificant contrasts from the main LME model			
	Cross-sectional contrasts			Longitudinal contrast
	AN-TP1 vs. HC	AN-TP2 vs. HC	RecAN vs. HC	AN at TP2 vs. TP1
Ant. sup. hypothalamus lh	n/a	68.00	37.06	n/a
Ant. sup. hypothalamus rh	n/a	82.82	34.72	n/a
Ant. inf. hypothalamus lh	12.21	55.25	53.12	42.02
Ant. inf. hypothalamus rh	n/a	45.66	38.69	n/a
Sup. tub. hypothalamus lh	n/a	n/a	10.92	16.59
Sup. tub. hypothalamus rh	n/a	36.55	24.07	n/a
Inf. tub. hypothalamus lh	n/a	14.12	35.19	n/a
Inf. tub. hypothalamus rh	n/a	26.06	58.13	n/a
Post. hypothalamus lh	n/a	85.69	60.32	n/a
Post. hypothalamus rh	n/a	18.11	22.06	n/a
Basal forebrain lh	n/a	n/a	14.37	28.61
Basal forebrain rh	53.94	34.14	25.32	48.03
Fornix lh	n/a	93.08	57.00	n/a
Fornix rh	n/a	54.11	38.25	n/a
Nucleus accumbens lh	n/a	24.96	30.04	n/a
Nucleus accumbens rh	n/a	81.31	14.39	n/a

Abbreviations: AN, participants with acute anorexia nervosa in the acutely underweight state (TP1) and reassessed after short-term weight-restoration (TP2); ant., anterior; HC, healthy control participants; inf., inferior; lh, left hemisphere; post., posterior; RecAN, long-term weight-recovered participants with a history of AN; rh, right hemisphere; sup., superior; tub., tubular.

Bayesian-framework LME model analysis was conducted to complement nonsignificant group differences from the main LME model (Figure 2 in the main article). Bayesian LME model structure was identical to the main LME model, predicting volumetric change in subcortical limbic structures as a linear function of BMI-SDS change (Figure 2, SM 1.6) and covarying for linear and quadratic age effects and head size (estimated total intracranial volume). The Bayesian model used a weakly informative normal distribution as prior, applying automated scale adjustment while fitting[10]. Markov Chain Monte Carlo posterior sampling was performed based on 10 randomly initialized chains of 5,000 iterations each and a warm-up period of 1,000 iterations[10]. For *nonsignificant* group contrasts from the main LME model exclusively (Figure 2), Bayes factors (BF_{01}) in favor of the null hypothesis (i.e., absence of group differences) are reported, obtained from post-hoc pairwise contrasts within the Bayesian LME model. Thresholding: BF_{01} >3 indicates substantial evidence in favor of the null hypothesis[11]. For significant group contrasts from the main LME model, Bayes factors were not computed (n/a).

Table S7. Supplementary LME models for longitudinal associations of subcortical limbic structures with demographic and clinical characteristics

Demographic associations

**Modified LME model**	**A.** Subcortical limbic structure volume = Intercept$_{Random}$ + Δ_{AN} + Δ_{RecAN} + A_{AN}*(BMI-SDS$_{TP(x)}$–BMI-SDS$_{TP1}$) + B_{AN}*(Age group *X* [BMI-SDS$_{TP(x)}$–BMI-SDS$_{TP1}$]) + C*Age + CC*Age$^{2\perp}$ + D*eTIV						
**Subcortical limbic structure**	**Pred. of interest**	***β***	**se**	**t**	**p**	**q**	**d**
Ant. sup. hypothalamus lh	Age group (<18 vs. ≥18 years) *X* ΔBMI-SDS	-0.13	0.23	-0.59	0.556	0.908	0.09
Ant. sup. hypothalamus rh		-0.10	0.25	-0.41	0.681	0.908	0.06
Ant. inf. hypothalamus rh		0.05	0.23	0.24	0.811	0.956	0.04
Sup. tub. hypothalamus rh		0.11	0.73	0.16	0.876	0.956	0.02
Inf. tub. hypothalamus lh		-1.23	0.61	-2.02	0.046	0.273	0.33
Inf. tub. hypothalamus rh		-0.47	0.63	-0.75	0.454	0.908	0.12
Post. hypothalamus lh		-0.75	0.64	-1.18	0.239	0.574	0.18
Post. hypothalamus rh		-1.03	0.75	-1.38	0.170	0.510	0.21
Fornix lh		-2.57	1.75	-1.47	0.143	0.510	0.27
Fornix rh		-4.85	2.00	-2.42	0.017	0.202	0.44
Nucleus accumbens lh		-1.67	3.86	-0.43	0.665	0.908	0.06
Nucleus accumbens rh		0.03	3.60	0.01	0.993	0.993	<0.01

Clinical associations

**Modified LME model**	**B.** Subcortical limbic structure volume = Intercept$_{Random}$ + Δ_{AN} + Δ_{RecAN} + A_{AN}*(BMI-SDS$_{TP(x)}$–BMI-SDS$_{TP1}$) + B_{AN}*(DOI *X* [BMI-SDS$_{TP(x)}$–BMI-SDS$_{TP1}$]) + C*Age + CC*Age$^{2\perp}$ + D*eTIV							**C.** Subcortical limbic structure volume = Intercept$_{Random}$ + Δ_{AN} + Δ_{RecAN} + A_{AN}*(BMI-SDS$_{TP(x)}$–BMI-SDS$_{TP1}$) + B_{AN}*(AN-subtype *X* [BMI-SDS$_{TP(x)}$–BMI-SDS$_{TP1}$]) + C*Age + CC*Age$^{2\perp}$ + D*eTIV						
**Subcortical limbic structure**	**Pred. of interest**	***β***	**se**	**t**	**p**	**q**	**d**	**Pred. of interest**	***β***	**se**	**t**	**p**	**q**	**d**
Ant. sup. hypothalamus lh	DOI *X* ΔBMI-SDS	<0.01	0.01	-0.17	0.866	0.866	0.03	AN-subtype (binge/purge vs. restrictive) *X* ΔBMI-SDS	0.05	0.28	0.17	0.868	0.933	0.03
Ant. sup. hypothalamus rh		<0.01	0.01	0.48	0.635	0.762	0.07		-0.03	0.31	-0.08	0.933	0.933	0.01
Ant. inf. hypothalamus rh		0.01	0.01	0.86	0.388	0.758	0.13		0.06	0.28	0.21	0.833	0.933	0.03
Sup. tub. hypothalamus rh		0.04	0.02	1.53	0.127	0.553	0.23		0.12	0.91	0.13	0.897	0.933	0.02
Inf. tub. hypothalamus lh		-0.03	0.02	-1.68	0.095	0.553	0.27		-0.29	0.77	-0.38	0.705	0.933	0.06
Inf. tub. hypothalamus rh		-0.01	0.02	-0.60	0.547	0.762	0.10		0.35	0.78	0.45	0.653	0.933	0.07

Post. hypothalamus lh		0.02	0.02	0.77	0.442	0.758	0.11		0.47	0.80	0.59	0.556	0.933	0.09
Post. hypothalamus rh		-0.03	0.02	-1.28	0.203	0.553	0.20		-0.10	0.93	-0.11	0.916	0.933	0.02
Fornix lh		-0.08	0.06	-1.33	0.186	0.553	0.25		-0.33	2.18	-0.15	0.879	0.933	0.03
Fornix rh		-0.02	0.07	-0.37	0.714	0.779	0.07		-5.02	2.48	-2.02	0.045	0.271	0.37
Nucleus accumbens lh		-0.15	0.13	-1.20	0.230	0.553	0.18		-11.48	4.66	-2.46	0.015	0.177	0.37
Nucleus accumbens rh		-0.06	0.12	-0.54	0.592	0.762	0.08		-3.53	4.45	-0.79	0.429	0.933	0.12
**Modified LME model**	**D.** Subcortical limbic structure volume = $\text{Intercept}_{Random} + \Delta_{AN} + \Delta_{RecAN} + A_{AN}*(\text{BMI-SDS}_{TP(x)}–\text{BMI-SDS}_{TP1}) + B_{AN}*(\text{EDI-2 core}_{TP(x)}–\text{EDI-2 core}_{TP1}) + C*Age + CC*Age^{2\perp} + D*eTIV$							**E.** Subcortical limbic structure volume = $\text{Intercept}_{Random} + \Delta_{AN} + \Delta_{RecAN} + A_{AN}*(\text{BMI-SDS}_{TP(x)}–\text{BMI-SDS}_{TP1}) + B_{AN}*(\text{BDI-II total}_{TP(x)}–\text{BDI-II total}_{TP1}) + C*Age + CC*Age^{2\perp} + D*eTIV$					
**Subcortical limbic structure**	**Pred. of interest**	**β**	**se**	**t**	**p**	**q**	**d**	**Pred. of interest**	**β**	**se**	**t**	**p**	**q**	**d**
Ant. sup. hypothalamus lh	ΔEDI-2 core	-0.01	0.04	-0.30	0.761	0.913	0.05	ΔBDI-II total	<0.01	0.02	-0.16	0.876	0.959	0.02
Ant. sup. hypothalamus rh		-0.01	0.04	-0.24	0.808	0.913	0.03		<0.01	0.02	-0.05	0.959	0.959	<0.01
Ant. inf. hypothalamus rh		-0.02	0.04	-0.46	0.648	0.913	0.07		0.02	0.02	0.78	0.437	0.928	0.12
Sup. tub. hypothalamus rh		-0.12	0.13	-0.98	0.329	0.564	0.15		-0.04	0.07	-0.61	0.541	0.928	0.09
Inf. tub. hypothalamus lh		0.15	0.10	1.44	0.152	0.365	0.24		0.04	0.06	0.61	0.542	0.928	0.10
Inf. tub. hypothalamus rh		0.01	0.11	0.12	0.906	0.913	0.02		-0.02	0.06	-0.39	0.694	0.959	0.06
Post. hypothalamus lh		0.20	0.11	1.80	0.073	0.365	0.27		0.08	0.06	1.31	0.191	0.875	0.20
Post. hypothalamus rh		0.20	0.13	1.58	0.117	0.365	0.25		0.09	0.07	1.23	0.219	0.875	0.20
Fornix lh		0.80	0.29	2.72	0.008	0.091	0.51		0.11	0.17	0.65	0.517	0.928	0.12
Fornix rh		0.54	0.35	1.52	0.130	0.365	0.28		-0.04	0.20	-0.22	0.830	0.959	0.04
Nucleus accumbens lh		0.75	0.65	1.15	0.250	0.500	0.17		0.49	0.37	1.32	0.190	0.875	0.20
Nucleus accumbens rh		-0.07	0.61	-0.11	0.913	0.913	0.02		-0.04	0.35	-0.12	0.906	0.959	0.02

Abbreviations: AN, participants with acute anorexia nervosa in the acutely underweight state (TP1) and reassessed after short-term weight-restoration (TP2); ant., anterior; BDI-II total, total score of Beck Depression Inventory-II; BMI-SDS, body mass index-standard deviation score; DOI, duration of illness; EDI-2 core, averaged score comprising the core subscales drive for thinness, body dissatisfaction, and bulimia of Eating Disorder Inventory-2; eTIV, estimated total intracranial volume; inf., inferior; lh, left hemisphere; post., posterior; RecAN, long-term weight-recovered participants with a history of AN; rh, right hemisphere; sup., superior; TP(x), study time point in AN participants (either TP1 or TP2); tub., tubular.

Modifications of the main LME model (SM 1.6, Figure 2 in the main article) to investigate longitudinal associations of change scores of further demographic and clinical variables (predictors of interest) and volumes of subcortical limbic structures over the course of short-term weight-restoration in AN (only those volumes with significant change in AN-TP2

vs. AN-TP1 [Figure 2] were analyzed; models included all study groups). As model statistics for each predictor of interest, unstandardized coefficient ($\boldsymbol{\beta}$), its standard error (se), t-value, unadjusted p-value (two-tailed), FDR-q (FDR adjustment across all tested volumes per demographic/clinical variable), and Cohen's d are stated.

SM 3 Supplementary figures

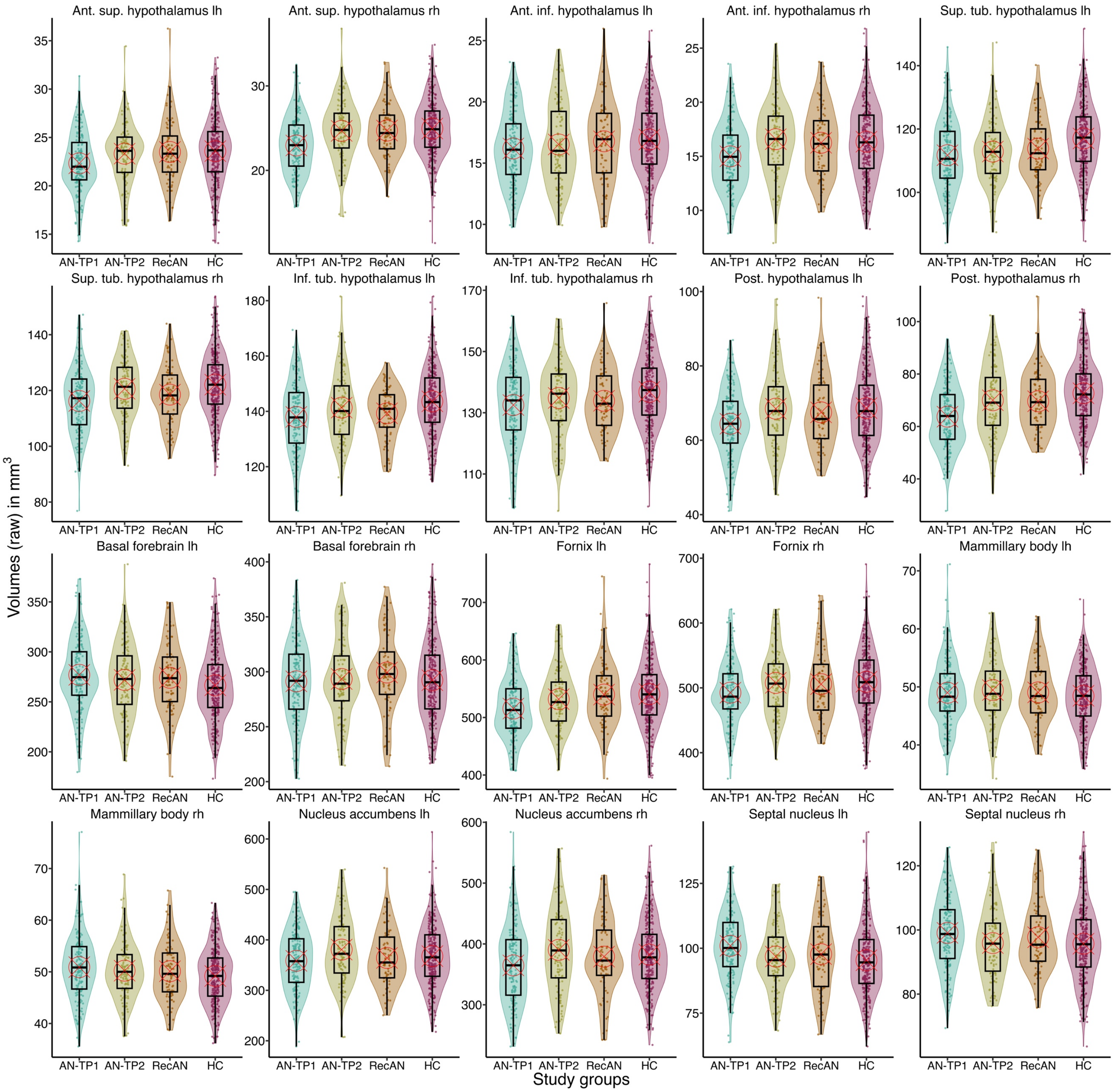

Figure S1. Combined plots for raw volumes of subcortical limbic structures

Abbreviations: AN, participants with acute anorexia nervosa in the acutely underweight state (TP1) and reassessed after short-term weight-restoration (TP2); ant., anterior; HC, healthy control participants; inf., inferior; lh, left hemisphere; post., posterior; RecAN, long-term weight-recovered participants with a history of AN; rh, right hemisphere; sup., superior; tub., tubular.

Plots visualize individual raw subcortical limbic structure volumes in each study group (points), their distribution (black box plots, red crosses indicating study group-wise means), and probability density (colored violin plots).

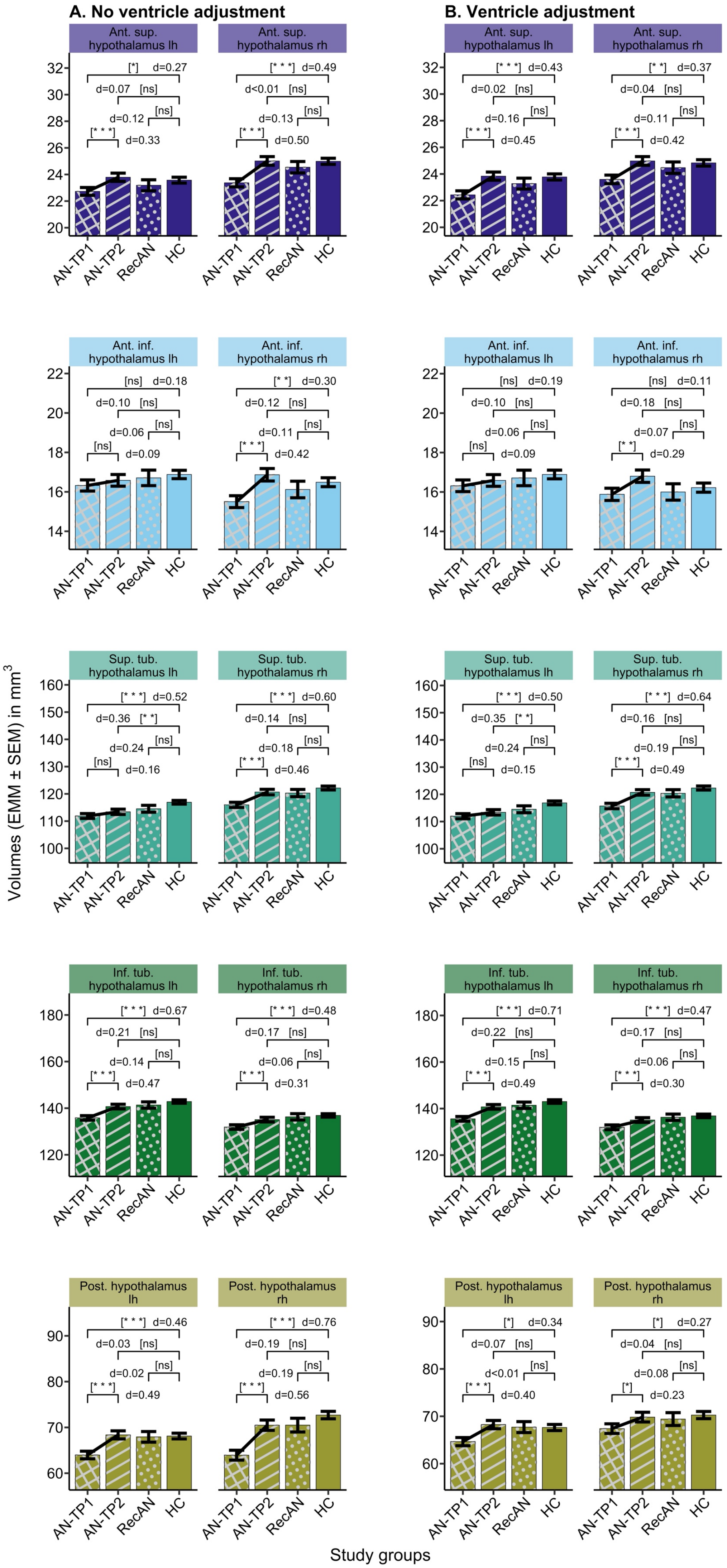

Figure S2. Exploratory analysis to compare group differences in subcortical limbic structures without vs. with adjustment for adjacent ventricle volumes

Abbreviations: AN, participants with acute anorexia nervosa in the acutely underweight state (TP1) and reassessed after short-term weight-restoration (TP2); ant., anterior; HC, healthy control participants; inf., inferior; lh, left hemisphere; post., posterior; RecAN, long-term weight-recovered participants with a history of AN; rh, right hemisphere; sup., superior; tub., tubular.

A. Main LME model (SM 1.6, Figure 2 in the main article) for group differences in subcortical limbic structures (all structures analyzed including mammillary bodies and septal nuclei).

B. Modification of the main LME model additionally covarying for adjacent ventricle volumes (all subcortical limbic structures analyzed including mammillary bodies and septal nuclei).

Bar plots display model estimates: estimated marginal mean (EMM) ± standard error of the mean (SEM) of individual volumes of subcortical limbic structures in each study group. Significance levels (FDR adjustment across all contrasts and all volumes per model A./B.): [***], FDR-q<0.001; [**], FDR-q<0.01; [*], FDR-q<0.05; [ns], nonsignificant.

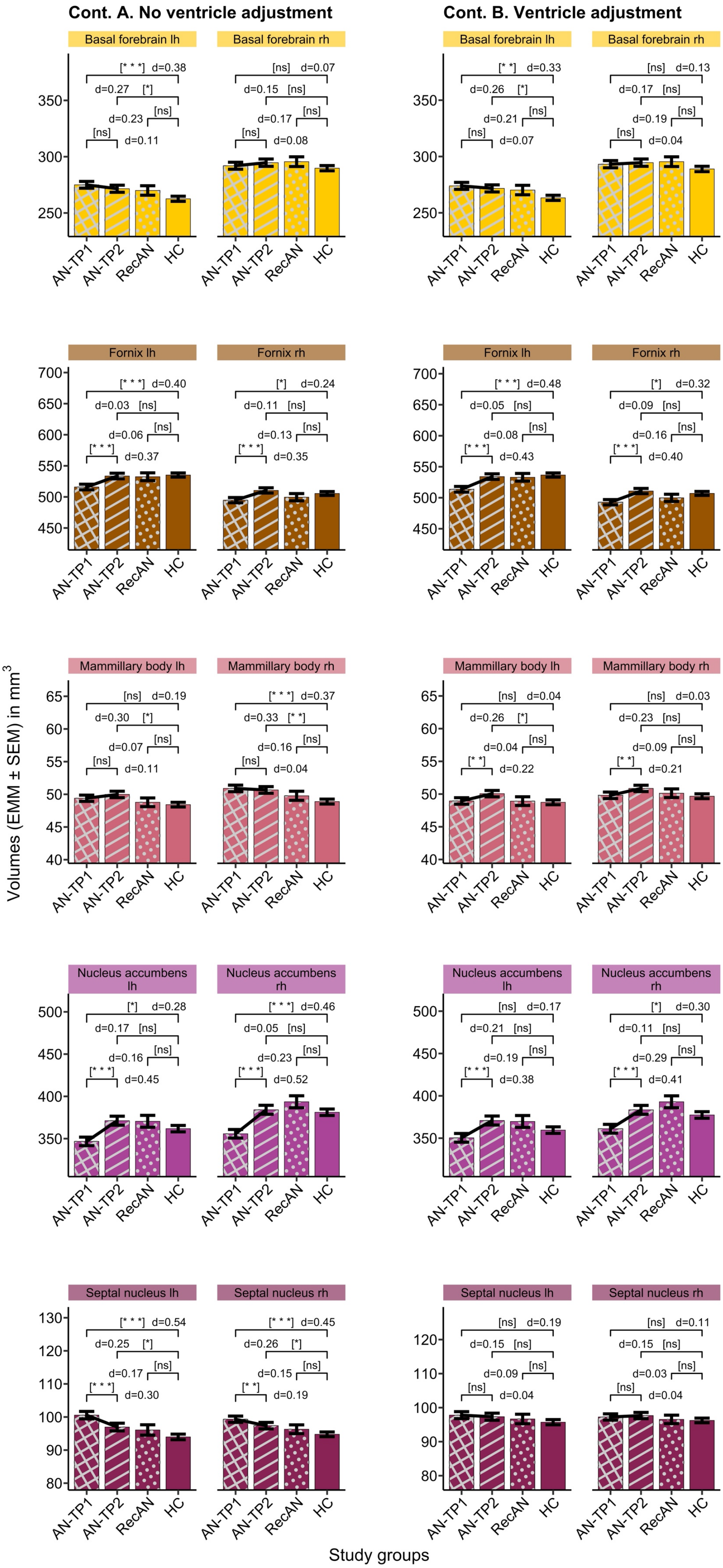

Figure S2. Continued

For bilateral mammillary bodies and septal nuclei, discrepancies between models **A.** (without ventricle adjustment) and **B.** (with ventricle adjustment) were detected indicating potential segmentation bias. Thus, both structures were excluded from main analyses (SM 1.6).

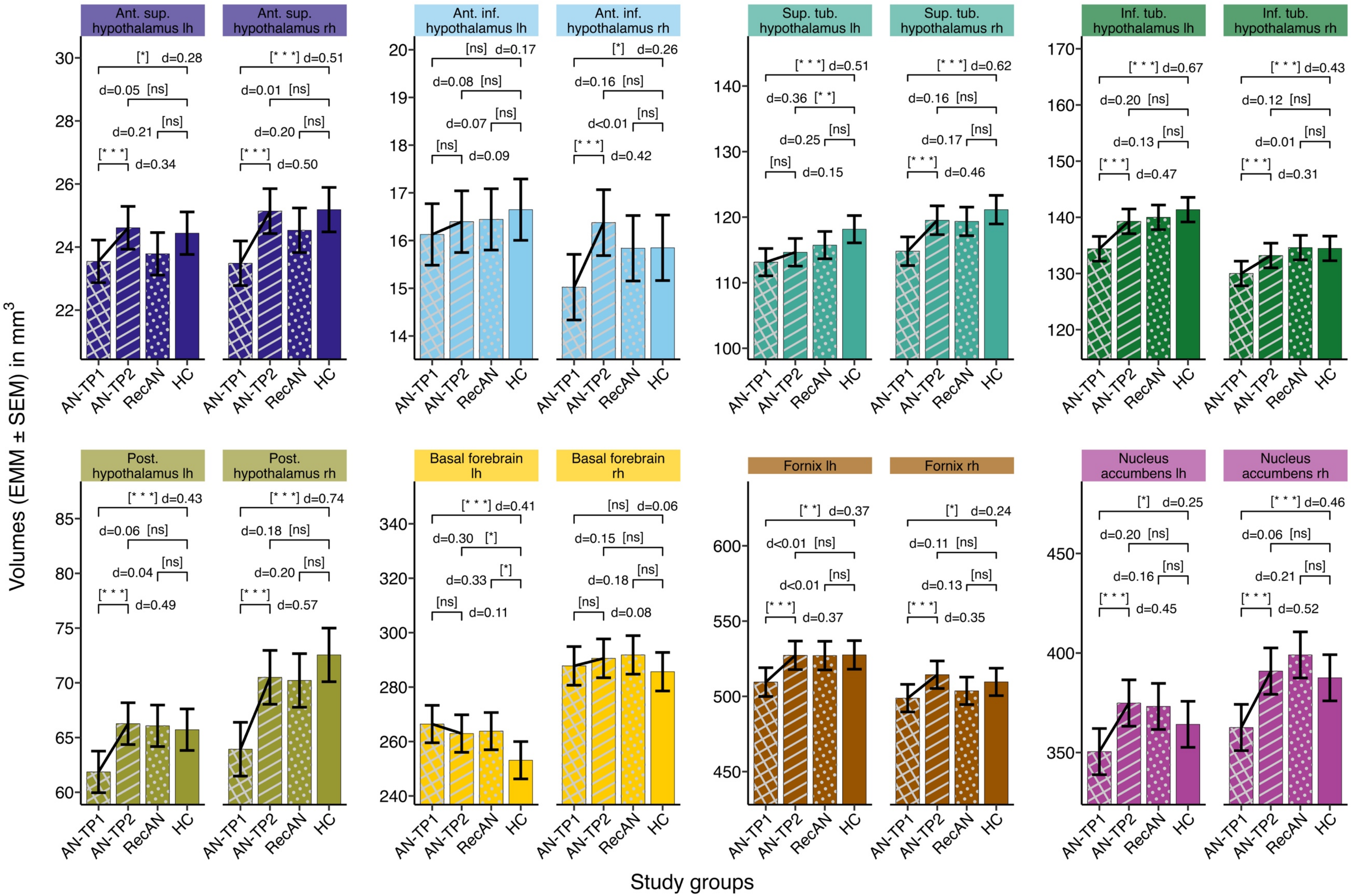
Ant. sup. hypothalamus lh
Ant. sup. hypothalamus rh
Ant. inf. hypothalamus lh
Ant. inf. hypothalamus rh
Sup. tub. hypothalamus lh
Sup. tub. hypothalamus rh
Inf. tub. hypothalamus lh
Inf. tub. hypothalamus rh
Post. hypothalamus lh
Post. hypothalamus rh
Basal forebrain lh
Basal forebrain rh
Fornix lh
Fornix rh
Nucleus accumbens lh
Nucleus accumbens rh
AN-TP1
AN-TP2
RecAN
HC
Volumes (EMM ± SEM) in mm3
Study groups

Figure S3. Modified LME model for group differences in subcortical limbic structures accounting for cigarette smoking, co-existing psychiatric disorders, and psychoactive medication

Abbreviations: AN, participants with acute anorexia nervosa in the acutely underweight state (TP1) and reassessed after short-term weight-restoration (TP2); ant., anterior; HC, healthy control participants; inf., inferior; lh, left hemisphere; post., posterior; RecAN, long-term weight-recovered participants with a history of AN; rh, right hemisphere; sup., superior; tub., tubular.

Modification of the main LME model (SM 1.6, Figure 2 in the main article) to reproduce group differences in subcortical limbic structures, additionally adjusting for dichotomous covariates: current cigarette smoking (yes if >1 cigarette/day on average), presence of co-existing psychiatric conditions in AN and RecAN (yes if psychiatric conditions other than AN reported, e.g., depressive disorder, anxiety disorder, obsessive-compulsive disorder, post-traumatic stress disorder, or addiction), and current psychoactive medication intake in AN and RecAN (yes if prescribed antidepressants [SSRIs and mirtazapine, see SM 1.1]; all other psychoactive drugs a-priori excluded). **Effects of all three covariates were nonsignificant for all subcortical limbic structures.** Bar plots display model estimates: estimated marginal mean (EMM) ± standard error of the mean (SEM) of individual volumes of subcortical limbic structures in each study group. FDR-adjusted significance (FDR adjustment across all contrasts and all volumes) and effect size estimate Cohen's d are presented for contrasts of interest (SM 1.6). Significance levels: [***], FDR-q<0.001; [**], FDR-q<0.01; [*], FDR-q<0.05; [ns], nonsignificant. Please consult Table 1 in the main article for descriptive details on cigarette smoking, psychiatric comorbidities, and psychoactive medication. Please note that other potentially confounding demographic characteristics *(intelligence quotient, socioeconomic status, and hand preference)* were not analyzed as covariates in the confirmatory LME model, since there were no differences between study groups in these characteristics (see Table 1).

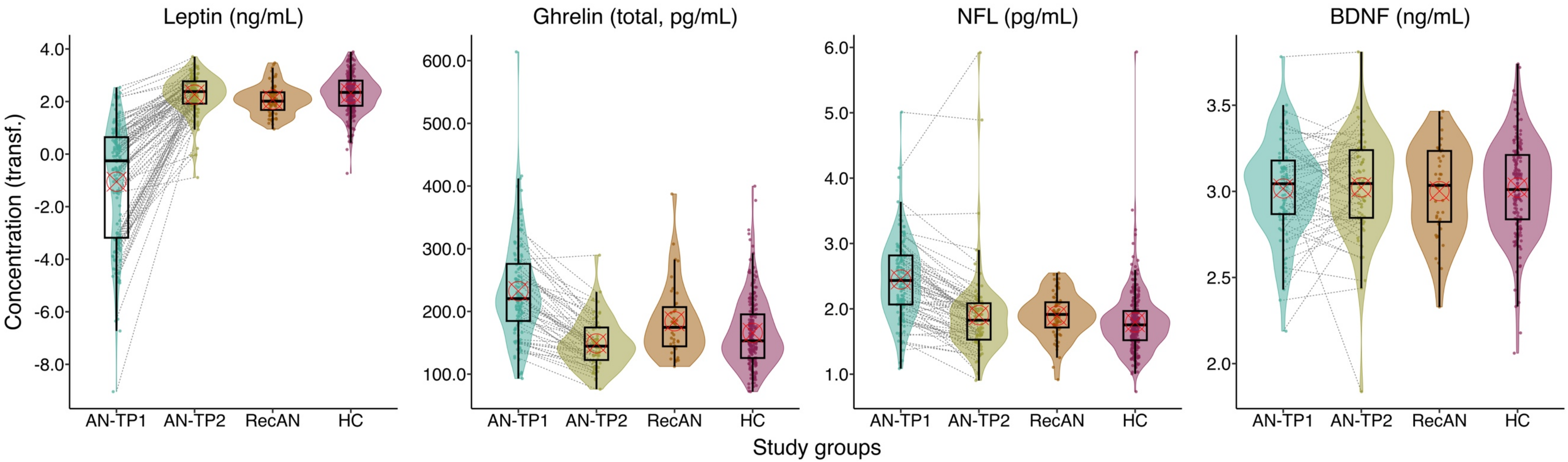

Figure S4. Blood-based protein marker concentrations in each study group

Abbreviations: AN, participants with acute anorexia nervosa in the acutely underweight state (TP1) and reassessed after short-term weight-restoration (TP2); BDNF, brain-derived neurotrophic factor; HC, healthy control participants; NFL, neurofilament light; RecAN, long-term weight-recovered participants with a history of AN; transf., transformed. Combined plots visualize individual transformed concentrations of leptin ($\log_e$-transformed), ghrelin ($x^{0.75}$-transformed), NFL ($\log_e$-transformed), and BDNF ($\log_e$-transformed) in each study group (colored points), their distribution (black box plots and red crosses [indicating study group-wise means]), and probability density (colored violin plots). Individual change scores AN-TP2-AN-TP1 of markers are indicated by dashed gray lines (one line per AN participant).

Cross-correlations (Pearson) between (transformed) marker concentrations in patients with AN (AN-TP1, adjusted for BMI-SDS) showed that leptin was significantly and inversely associated with NFL ($r=-0.24$, $p=0.006$). All other correlations between markers were nonsignificant (leptin-ghrelin: $r=-0.06$, $p=0.529$; leptin-BDNF: $r=0.16$, $p=0.136$; ghrelin-NFL: $r=0.10$, $p=0.302$; ghrelin-BDNF: $r=-0.02$, $p=0.835$; NFL-BDNF: $r=-0.08$, $p=0.480$).

References

1. Fichter M, Quadflieg N. *Strukturiertes Inventar Für Anorektische Und Bulimische Eßstörungen Nach DSM-IV Und ICD-10 (SIAB).* 1st ed. Hogrefe; 1999.

2. Fichter M, Quadflieg N. The structured interview for anorexic and bulimic disorders for DSM-IV and ICD-10 (SIAB-EX): reliability and validity. *Eur psychiatr*. 2001;16(1):38-48. doi:10.1016/S0924-9338(00)00534-4

3. Boss J, Mukherjee B, Ferguson KK, et al. Estimating Outcome-Exposure Associations when Exposure Biomarker Detection Limits vary Across Batches: *Epidemiology*. 2019;30(5):746-755. doi:10.1097/EDE.0000000000001052

4. Rubin DB. *Multiple Imputation for Nonresponse in Surveys*. Wiley; 1987.

5. Kong X, Postema MC, Guadalupe T, et al. Mapping brain asymmetry in health and disease through the ENIGMA consortium. *Hum Brain Mapp*. Published online May 18, 2020:hbm.25033. doi:10.1002/hbm.25033

6. Sämann PG, Iglesias JE, Gutman B, et al. FreeSurfer-based segmentation of hippocampal subfields: A review of methods and applications, with a novel quality control procedure for ENIGMA studies and other collaborative efforts. *Hum Brain Mapp*. Published online December 27, 2020:hbm.25326. doi:10.1002/hbm.25326

7. Ma Z, Reich DS, Dembling S, Duyn JH, Koretsky AP. Outlier detection in multimodal MRI identifies rare individual phenotypes among more than 15,000 brains. *Human Brain Mapping*. 2022;43(5):1766-1782. doi:10.1002/hbm.25756

8. Fischl B. FreeSurfer. *NeuroImage*. 2012;62(2):774-781. doi:10.1016/j.neuroimage.2012.01.021

9. Benjamini Y, Hochberg Y. Controlling the False Discovery Rate: A Practical and Powerful Approach to Multiple Testing. *Journal of the Royal Statistical Society: Series B (Methodological)*. 1995;57(1):289-300. doi:10.1111/j.2517-6161.1995.tb02031.x

10. Goodrich B, Gabry J, Ali I, Brilleman S. rstanarm: Bayesian applied regression modeling via Stan. Published online 2020. https://mc-stan.org/rstanarm

11. Kass RE, Raftery AE. Bayes Factors. *Journal of the American Statistical Association*. 1995;90(430):773-795. doi:10.1080/01621459.1995.10476572
